# 6-Shogaol Overcomes Gefitinib Resistance via ER Stress in Ovarian Cancer Cells

**DOI:** 10.3390/ijms24032639

**Published:** 2023-01-30

**Authors:** Tae Woo Kim, Hee Gu Lee

**Affiliations:** 1 Department of Biopharmaceutical Engineering, Dongguk University-WISE, Gyeongju 38066, Republic of Korea; 2 Immunotherapy Research Center, Korea Research Institute of Bioscience and Biotechnology, Daejeon 34141, Republic of Korea

**Keywords:** 6-shogaol, ER stress, gefitinib, Nox4, resistance

## Abstract

In women, ovary cancer is already the fifth leading cause of mortality worldwide. The use of cancer therapies, such as surgery, radiotherapy, and chemotherapy, may be a powerful anti-cancer therapeutic strategy; however, these therapies still have many problems, including resistance, toxicity, and side effects. Therefore, natural herbal medicine has the potential to be used for cancer therapy because of its low toxicity, fewer side effects, and high success. This study aimed to investigate the anti-cancer effect of 6-shogaol in ovarian cancer cells. 6-shogaol induces ER stress and cell death via the reduction in cell viability, the increase in LDH cytotoxicity, caspase-3 activity, and Ca^2+^ release, and the upregulation of GRP78, p-PERK, p-eIF2α, ATF-4, CHOP, and DR5. Moreover, 6-shogaol treatment medicates endoplasmic reticulum (ER) stress and cell death by upregulating Nox4 and releasing ROS. The knockdown of Nox4 in ovarian cancer cells inhibits ER stress and cell death by blocking the reduction in cell viability and the enhancement of LDH cytotoxicity, caspase-3 activity, Ca^2+^, and ROS release. In gefitinib-resistant ovarian cancer cells, A2780R and OVCAR-3R, 6-shogaol/gefitinib overcomes gefitinib resistance by inhibiting EMT phenomena such as the reduction in E-cadherin, and the increase in N-cadherin, vimentin, Slug, and Snail. Therefore, our results suggest that 6-shogaol exerts a potential anti-cancer effect in ovarian cancer and combination treatment with 6-shogaol and gefitinib may provide a novel anti-tumor therapeutic strategy in gefitinib-resistant ovarian cancer.

## 1. Introduction

Ovarian cancer is the fifth most common type of cancer among women and is one of the most common gynecologic cancer types [1]. Ovarian cancer is divided into three types such as epithelial ovarian carcinomas, germ cell tumors, and stromal cell tumors. Of these types, epithelial ovarian carcinomas are the most common [2]. Many researchers have repeatedly focused on ovarian cancer therapy, but it still has a resistance problem. Therefore, a tumor therapeutic strategy is needed that can overcome resistance and improve ovarian cancer therapy. 

The ER stress response plays a dual role in cell survival and cell death in various diseases, including cancer, diabetes, and cardiovascular disease [3]. ER stress induces cell death via the activation of the unfolded protein response (UPR) in the tumor environment, and targeting ER stress to overcome chemo-resistance has recently provided a powerful and promising anti-cancer strategy such as through combinatory therapy [4]. Reactive oxygen species (ROS) contribute to many diseases, and ROS-mediated stresses regulate tumor growth and are an important factor inducing cell death via the activation of ER stress [5]. Traditional medicine derivative compounds exert an anti-cancer effect via ER stress in various cancer types [6]. *Paris polyphylla* derivative polyphyllin D induces ER stress and cell death via GRP78, CHOP, and caspase-3 cleavage in NSCLC cells NCI-H460, and *Saussurea lappa* and *Auckland lappa* derivative dehydrocostuslactone mediates ER stress and cell death by activating PERK-CHOP and IRE-1-JNK signaling and inducing ROS and Ca^2+^ release in NSCLC cells A549 and NCI-H460 [7,8]. Guggulsterone extracted from *Commiphora mukul* induces apoptotic cell death via the upregulation of GRP78, PERK, p-JNK, CHOP, and DR5 in Hep3B cells, whereas CHOP knockdown inhibits an anti-cancer effect of guggulsterone [9]. In recent anti-cancer therapy, epidermal growth factor receptor tyrosine kinase inhibitors (EGFR-TKIs), such as gefitinib and erlotinib, have been used as a powerful therapeutic strategy to block tumor growth by binding certain parts of EGFR and have improved the survival rates in tumor patients [10,11]. Phase II trials of gefitinib in ovarian cancer reported low toxicity and promising clinical results by inhibiting the phosphorylation of EGFR [12]. Though having promising potential, representative EGFR inhibitor gefitinib has frequently induced chemo-resistance in many clinical studies, and thus it has failed to improve the survival rate in tumor patients [13]. To overcome gefitinib resistance in the tumor microenvironment, combination therapy has been suggested as an alternative strategy to ensure effective cancer therapy [14,15]. Moreover, to overcome resistance to gefitinib treatment, we need a deeper understanding of the molecular mechanism and clinical predictors in gefitinib-resistant ovarian cancer [16]. 

Ginger (*Zingiber officinale Roscoe*) has been reported to have anti-cancer, anti-oxidant, and anti-inflammatory effects to treat various disease types, including cancer, arthritis, rheumatism, and indigestion [17,18]. Ginger is abundant in constituents, including phenolic and terpene compounds, and has major compounds such as 6-shogaol, 6-gingerol, 8-gingerol, and 10-gingerol [19]. Major compounds extracted from ginger exert a potential anti-cancer effect by inhibiting the phosphorylation of EGFR (epidermal growth factor receptor) in various cancer types [20]. The representative first-generation EGFR-TKI (tyrosine kinase inhibitor) gefitinib induces apoptotic cell death via the intracellular Ca2+ and ROS release, the upregulating of Nox2 or Nox4, and the ER stress response in various cancer cell types [21,22]. Gefitinib treatment acquired resistance in various mechanisms such as EGFR mutation, the amplification of tyrosine kinase receptors, and the EMT phenomenon [23,24,25]. In addition to the general mechanism to acquire gefitinib resistance, an excessive ER stress response by combination therapy might be an alternative strategy via intracellular Ca2+ release and ROS production to overcome gefitinib resistance in cancer therapy [26,27]. Recently, many studies have reported that combination therapy with anti-cancer drugs exerts a powerful anti-cancer effect by targeting ER stress in gefitinib-resistant cancer cells [28]. In gefitinib-sensitive and -resistant NSCLC cells, HCC827 and HCC827GR, licochalcone extracted from Glycyrrhiza inflata blocks cell viability and colony formation and induces G2/M cell cycle arrest, apoptosis, ER stress, ROS release, MMP loss, and caspase activity [29]. Honokiol, a natural chemical isolated from *Magnolia Officinalis*, mediates the degradation of Hsp90, the inhibition of Akt and ERK, and the activation of ER stress in gefitinib-sensitive and gefitinib-resistant NSCLC cells [30]. In pancreatic cancer cell lines BxPC3 and PANC-1, combined treatment with gefitinib and autophagy inhibitors, such as clarithromycin, azithromycin, and EM900, enhances apoptosis and cell death through the ER stress response [31]. Numerous studies have shown that ER stress suppresses tumor growth and overcomes EGFR-mediated resistance by inhibiting the phosphorylation of EGFR. In addition, many reports have indicated that 6-shogaol induces apoptotic cell death by inhibiting the phosphorylation of EGFR in various cancer cell types. Based on these results, if 6-shogaol induces apoptosis through the ER stress response in ovarian cancer cells, 6-shogaol alone or 6-shogaol plus gefitinib may be a potential novel therapeutic strategy in gefitinib-resistant models.

Therefore, we studied whether ginger bioactive compound 6-shogaol exerts an anti-cancer effect via the ER stress response in ovarian cancer cell lines. In addition, we examined whether a combined treatment with 6-shogaol and gefitinib can overcome gefitinib resistance via the activation of ER stress and the suppression of the EMT process in gefitinib-resistant ovarian cancer cells. 

## 2. Results

### 2.1. 6-shogaol Induces an Anti-cancer Effect in Ovarian Cancer Cells

**Figure 1:**
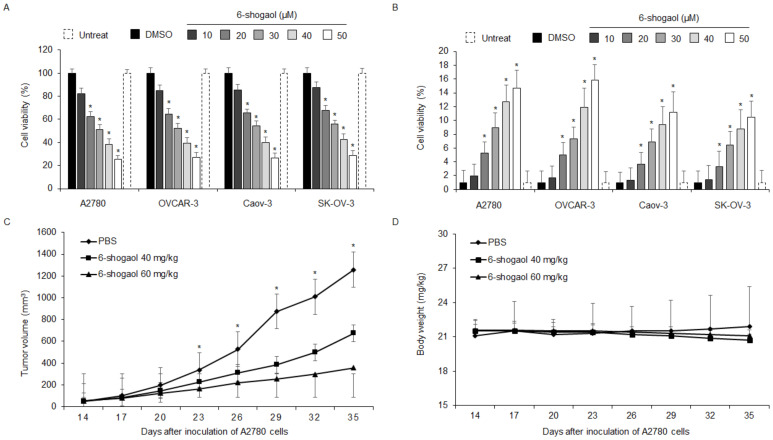
Anti-cancer effects of 6-shogaol in ovarian cancer cell lines in dose-dependent manner. (**A**,**B**) Cell viability and LDH cytotoxicity of a dose-dependent 6-shogaol, in ovarian cancer cell lines, including A2780, OVCAR-3, Caov-3, and SK-OV-3, measured using the WST-1 assay and LDH assay on 96-well plates; *, *p* < 0.05. (**C**,**D**) A2780 cells (1 × 10^7^) were implanted (sc) into the thigh on the right hind leg of nude mice (n=10/group). 6-shogaol (40 or 60 mg/kg) or PBS was administered (ip) once a day for two days. The longest (L) and shortest (W) axes of the tumors were measured, and the tumor volume (mm^3^) was calculated as LW2/2. Body weights of the A2780 tumor xenograft mice were determined twice a week during the experiment; *, *p* < 0.05.

**Figure 2:**
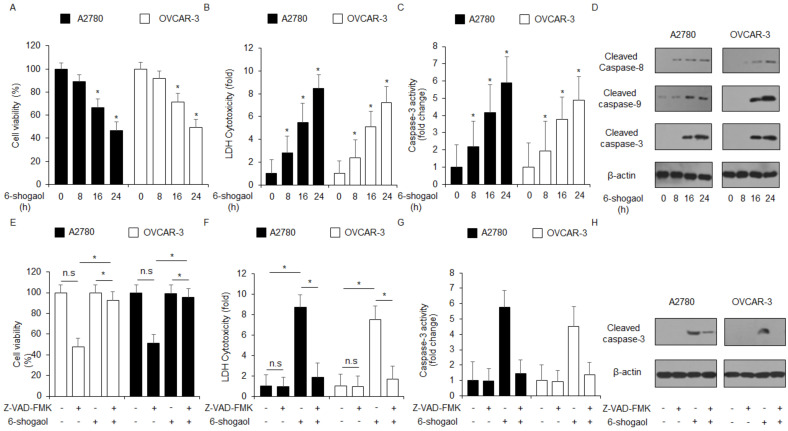
Anti-cancer effects of 6-shogaol in ovarian cancer cell lines in time-dependent manner. (**A**–**D**) 6-shogaol is Table 0. 16, and 24 h; 30 μM) and the WST-1 assay, LDH assay, and caspase-3 activity assays were performed. Western blotting analyses of cleaved caspase-3, -8, and -9 for the indicated times in 6-shogaol-treated A2780 and OVCAR-3 cells; *, *p* < 0.05. β-actin was used as a protein loading control. (**E**–**H**) The effect of Z-VAD-FMK (50 μM) and 6-shogaol treatment. A2780 and OVCAR-3 cells were pretreated with Z-VAD-FMK for 4 h and were subsequently treated with 6-shogaol (30 μM, 24 h). Cell viability was determined using the WST-1 assay; cell cytotoxicity was monitored using the LDH assay, and caspase-3 activity was assessed using the caspase-3 activity assay; *, *p* < 0.05. n.s = not significant. To identify caspase-3 cleavage, a Western blot assay was performed. β-actin was used as a protein loading control.

To identify the anti-cancer effect of 6-shogaol in four ovarian cancer cell lines, including A2780, OVCAR-3, Caov-3, and SK-OV-3, we used a WST-1 and LDH assay in a dose-dependent manner. A dose-dependent anti-cancer effect of 6-shogaol was observed in ovarian cancer cell lines (Figure 1A,B). To investigate the effects of 6-shogaol in vivo, an ovarian cancer xenograft mice model was established with A2780 cells. 6-shogaol at 40 mg/kg and 60 mg/kg indicated lower tumor volumes compared with the control (Figure 1C). The body weight of all groups was not significant (Figure 1D). To confirm the anti-cancer effect of 6-shogaol in a time-dependent manner, ovarian cancer cell lines A2780 and OVCAR-3 were treated by 6-shogaol for 8, 16, and 24 h. After these treatments, we performed the tests for cell viability, cell cytotoxicity, and caspase-3 activity using WST-1, LDH, and caspase-3 activity. 6-shogaol treatment inhibits cell viability and increases LDH production and caspase-3 activity in a time-dependent manner in both cell lines (Figure 2A–C). Western blotting analyses also indicated that 6-shogaol treatment mediates caspase 3, 8, and 9 cleavages (Figure 2D). To investigate if 6-shogaol treatment induces caspase-dependent apoptosis in both cells, we performed a pharmacological inhibitor experiment using caspase inhibitor Z-VAD-FMK. Z-VAD-FMK alone did not change cell viability, LDH cytotoxicity, and caspase-3 activity; however, 6-shogaol alone inhibited cell viability and increased LDH cytotoxicity and caspase-3 activity. The combined treatment with 6-shogaol and Z-VAD-FMK blocked, dramatically, the inhibition of cell viability and the increase in LDH cytotoxicity and caspase-3 activity (Figure 2E–G). Moreover, Western blotting analyses found that 6-shogaol in combination with Z-VAD-FMK suppressed caspase-3 cleavage compared with 6-shogaol alone (Figure 2H). Our finding indicated that 6-shogaol induces apoptosis and cell death in ovarian cancer cell lines. 

### 2.2. 6-shogaol Treatment Induces ER Stress and Cell Death in Ovarian Cancer Cells 

**Figure 3:**
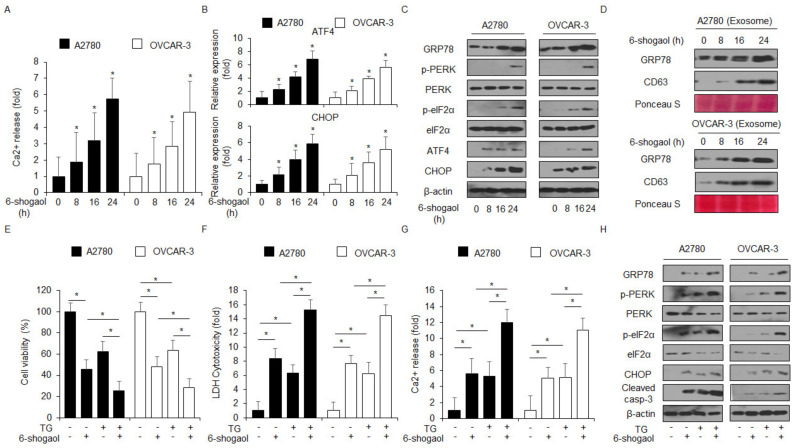
6-shogaol mediates ER stress response through intracellular Ca^2+^ generation. (**A**) A2780 and OVCAR-3 cells were treated with 6-shogaol (30 μM) in a time-dependent manner (0, 8, 16, and 24 h) and intracellular Ca^2+^ release was determined using an intracellular Ca^2+^ assay. (**B**) A2780 and OVCAR-3 cells were treated with 6-shogaol (30 μM) in a time-dependent manner (0, 8, 16, and 24 h) and mRNA levels of ATF4 and CHOP were investigated using real-time RT-PCR. (**C**) A2780 and OVCAR-3 cells were treated with 6-shogaol (30 μM) for the indicated times (0, 8, 16, and 24 h) and the activation of ER stress signaling, including GRP78, p-PERK, PERK, p-eIF2α, eIF2α, ATF4, and CHOP, was assessed using the Western blot assay. β-actin was used as a protein loading control. (**D**) A2780 and OVCAR-3 cells were treated with 6-shogaol (30 μM) for the indicated times (0, 8, 16, and 24 h) and exosomes were extracted from the cell culture media. Protein samples extracted from cell lysates and exosomes were quantified by Ponceau S staining. These samples were conducted by Western blotting using the ER stress marker, GRP78, and the exosome marker, CD63. (**E**–**H**) Cell viability, LDH cytotoxicity, and expression levels of GRP78, p-PERK, PERK, ATF4, CHOP, and cleaved caspase-3 levels were determined using WST-1 assay, LDH assay, and Western blotting analyses in thapsigargin (TG; 3 μM, 24 h) and 6-shogaol (30 μM, 24 h)-treated A2780 and OVCAR-3 cells; *, *p*<0.05.

The prolonged ER stress response induced by natural products exerts an anti-cancer effect by triggering the apoptosis signaling pathway, and thus, it could be a powerful anti-cancer strategy [32]. In addition, calcium (Ca^2+^) storage in ER plays a potential function in cell survival and cell death by regulating various intracellular signaling pathways [33]. Here, we showed that 6-shogaol induces intracellular Ca^2+^ release in a time-dependent manner in A2780 and OVCAR-3 cells (Figure 3A). To identify the mRNA expression of the ER stress response markers, such as ATF4 and CHOP, we performed real-time RT-PCR in 6-shogaol-mediated A2780 and OVCAR-3 cells. 6-shogaol increased the expression levels of ATF4 and CHOP to a greater extent than the control treatment in a time-dependent manner (Figure 3B). To study ER stress-related proteins, such as GRP78, p-PERK, PERK, p-eIF2α, eIF2α, ATF4, and CHOP, in 6-shogaol-treated A2780 and OVCAR-3 cells in a time-dependent manner, we carried out Western blotting analyses. 6-shogaol treatment upregulated time dependently the expression levels of GRP78, p-PERK, p-eIF2α, ATF4, and CHOP to a greater extent than the control treatment (Figure 3C). Furthermore, we investigated the ER stress master regulator GRP78 on the 6-shogaol-treated exosome fraction. Our results from the Western blot assay indicated that 6-shogaol treatment increases the expression levels of GRP78 and exosome marker CD63 (Figure 3D). To investigate whether ER stress was involved in 6-shogaol-treated cell death, we used thapsigargin (TG), an ER stress inducer, together with 6-shogaol to check cell viability, LDH cytotoxicity, Ca^2+^ production, and protein expression in the PERK signaling pathway. As shown in Figure 3E–H, 6-shogaol effectively induced the synergistic inhibition of cell viability, the increase in LDH and Ca^2+^ release, and the upregulation of GRP78, p-PERK, p-eIF2α, CHOP, and caspase-3 cleavage. 

### 2.3. Loss of GRP78 Suppresses 6-shogaol-Mediated Cell Death in Ovarian Cancer Cells 

**Figure 4:**
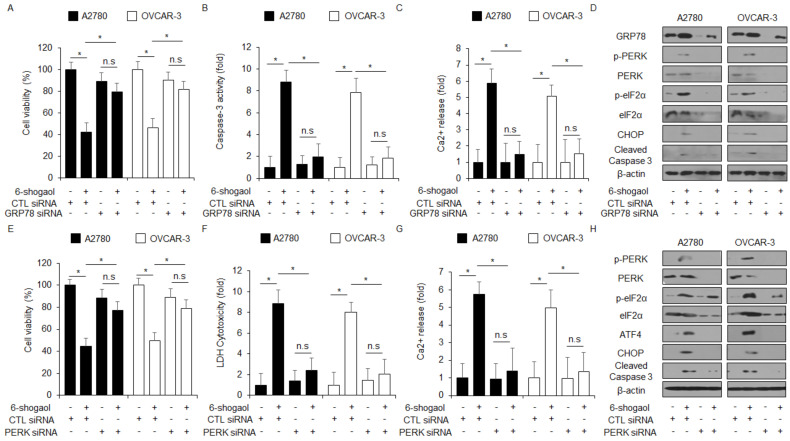
Inhibition of ER stress sensors suppressed by 6-shogaol caused cell death in ovarian cancer cells. (**A**–**C**) A2780 and OVCAR-3 cells were transfected with GRP78-specific siRNA in the presence or absence of 6-shogaol (30 μM, 24 h) and WST-1, caspase-3 activity, and intracellular Ca^2+^ assay were performed; *, *p*<0.05. (**D**) Western blot analysis of GRP78, p-PERK, PERK, p-eIF2α, eIF2α, ATF4, CHOP, and cleaved caspase-3 in 6-shogaol (30 μM, 24 h)-treated A2780 and OVCAR-3 cells was performed in the presence or absence of GRP78 siRNA (30 nM, 24 h). β-actin was used as protein loading controls. (**E**–**H**) After A2780 and OVCAR-3 cells were transfected with PERK (30 nM, 24 h), WST-1, LDH cytotoxicity assay, intracellular Ca^2+^ assay, and Western blotting analyses were performed with/without 6-shogaol (30 μM, 24 h) treatment.; *, *p* < 0.05. n.s = not significant. Western blotting was carried out to identify the ER stress-related genes p-PERK, PERK, p-eIF2α, eIF2α, ATF4, CHOP, and caspase-3 cleavage in 6-shogaol-treated PERK knockdown cells. β-actin was used as a protein loading control.

We determined the effect of GRP78 knockdown in 6-shogaol-treated ovarian cancer cells, A2780 and OVCAR-3. The suppression of GRP78 using specific siRNAs blocked the inhibition of cell viability and the increase in caspase-3 activity, Ca^2+^ release, and the upregulation of GRP78, p-PERK, p-eIF2α, eIF2α, CHOP, and caspase-3 cleavage in 6-shogaol-treated A2780 and OVCAR-3 cells (Figure 4A–D). To further confirm if 6-shogaol regulates ER stress and cell death, PERK-specific siRNAs were transfected into A2780 and OVCAR-3 cells, and then these cells were treated with 6-shogaol. The knockdown experiment of PERK suppressed the inhibition of cell viability and the enhancement of LDH and Ca^2+^ release, and the upregulation of GRP78, p-PERK, ATF4, CHOP, and caspase-3 cleavage more than control cells (Figure 4E–H). Our results indicated that the loss of GRP78 or PERK suppresses apoptotic cell death via the ER stress signaling pathway in 6-shogaol-treated ovarian cancer cells. 

### 2.4. CHOP Activation by 6-shogaol Treatment Induces Apoptotic Cell Death via DR4 in Ovarian Cancer Cells

**Figure 5:**
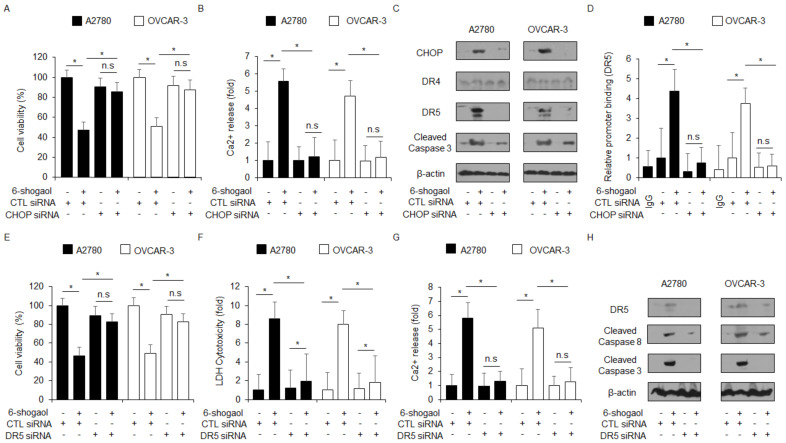
6-shogaol induces the binding of CHOP on DR5 promoter in ovarian cancer cells. (**A**–**D**) After A2780 and OVCAR-3 cells were transfected with CHOP siRNA (30 nM, 24 h), WST-1, intracellular Ca^2+^ assay, Western blotting analyses, and ChIP assay were performed with/without 6-shogaol (30 μM, 24 h) treatment.; *, *p* < 0.05. Western blotting analyses were carried out to determine the expression of CHOP, DR4, DR5, and caspase-3 cleavage in 6-shogaol-treated CHOP knockdown cells. β-actin was used as a protein loading control. (**E**–**H**) After A2780 and OVCAR-3 cells were transfected with DR5 (30 nM, 24 h), WST-1, LDH cytotoxicity assay, intracellular Ca^2+^ assay, and Western blotting analyses were performed with/without 6-shogaol (30 μM, 24 h) treatment.; *, *p* < 0.05. n.s = not significant. Western blotting was carried out to identify the expression of DR5, caspase-3, and caspase-8 cleavage in 6-shogaol-treated DR5 knockdown cells. β-actin was used as a protein loading control.

CHOP activation by ER stress induces ER stress and apoptotic cell death by binding on the DR4 or DR5 promoter [34,35]. To identify whether CHOP induced by 6-shogaol regulates DR4 and DR5, we performed a knockdown experiment for CHOP using specific siRNAs. The loss of CHOP blocked the inhibition of cell viability, the increase in Ca^2+^ release, and the upregulation of CHOP, DR5, and caspase-3 cleavage in 6-shogaol-treated A2780 and OVCAR-3 cells compared to control cells (Figure 5A–C). To investigate the further molecular mechanism, we performed a chromatin immunoprecipitation (ChIP) assay. As a result, 6-shogaol treatment binds CHOP on the DR5 promoter, whereas CHOP knockdown inhibits the binding of CHOP on the DR5 promoter in 6-shogaol-treated A2780 and OVCAR-3 cells (Figure 5D). To probe if DR5 regulates 6-shogaol-induced cell death, we performed a knockdown experiment for DR5 using specific siRNAs. Targeting DR5 blocked the inhibition of cell viability, the increase in LDH cytotoxicity and Ca^2+^ production, and the upregulation of DR5, caspase-8, and caspase-3 cleavage in 6-shogaol-treated A2780 and OVCAR-3 cells (Figure 5E–H).

### 2.5. 6-shogaol Mediates ER Stress and Cell Death via ROS Production in Ovarian Cancer Cells

**Figure 6:**
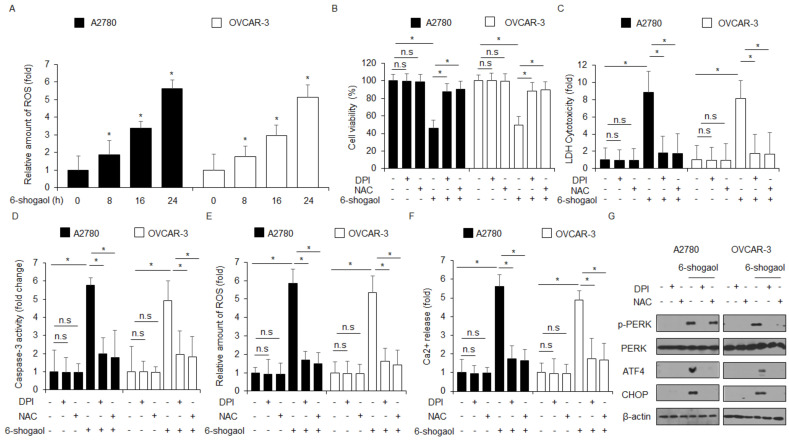
DPI and NAC suppresses cell death in 6-shogaol-treated ovarian cancer cells. (**A**) The fluorescence data indicate intracellular ROS production by DCFDA dye in 6-shogaol (0, 8, 16, and 24 h; 30 μM)-treated A2780 and OVCAR-3 cells. (**B**–**G**) A2780 and OVCAR-3 cells were pretreated with DPI (1 μM) and NAC (100 μM) for 4 h and subsequently treated with 6-shogaol (30 μM, 24 h). WST-1, LDH cytotoxicity assay, caspase-3 activity assay, ROS assay, intracellular Ca^2+^ assay, and Western blotting analyses were performed; *, *p* < 0.05. n.s = not significant. Western blot showing p-PERK, PERK, ATF4, and CHOP levels was carried out using these samples. β-actin was used as the protein loading control.

Excessive ROS release induces cell death in various cancer cell types, and thus, targeting ROS is a potential anti-cancer therapeutic strategy [36,37]. Moreover, a number of reports have suggested that ROS generation regulates cell death by inducing the ER stress signaling pathways [38]. To demonstrate whether 6-shogaol modulates the ROS release in ovarian cancer cells, the intracellular ROS assay was performed. 6-shogaol treatment mediates intracellular ROS generation in a time-dependent manner (Figure 6A). To study if ROS inhibitors DPI and NAC block 6-shogaol-treated ROS production and cell death in A2780 and OVCAR-3 cells, we carried out a WST-1 assay, LDH assay, caspase-3 activity assay, intracellular Ca^2+^ assay, and intracellular ROS assay. DPI + 6-shogaol or NAC + 6-shogaol treatment suppressed the inhibition of cell viability and the increase in LDH cytotoxicity, ROS generation, and Ca^2+^ release to a greater extent than 6-shogaol treatment alone (Figure 6B–F). In Western blot analyses, DPI + 6-shogaol or NAC + 6-shogaol treatment blocked the upregulation of p-PERK, ATF4, and CHOP expression in 6-shogaol-treated A2780 and OVCAR-3 cells compared to 6-shogaol alone (Figure 6G). 

### 2.6. 6-shogaol Induces ER Stress and Cell Death by Upregulating Nox4 in Ovarian Cancer Cells

**Figure 7:**
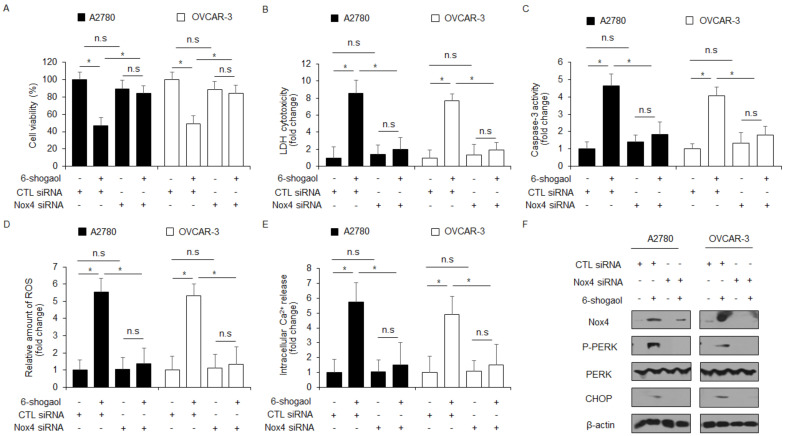
Nox4 regulates cell death and ER stress response in 6-shogaol-treated ovarian cancer cells. (**A**–**F**) A2780 and OVCAR-3 cells were transfected with Nox4 siRNAs and treated with 6-shogaol (30 μM, 24 h). WST-1 assay, LDH cytotoxicity assay, caspase-3 activity assay, intracellular ROS assay, and intracellular Ca^2+^ assay was performed along with Western blot analysis for Nox4, p-PERK, PERK, and CHOP; *, *p* < 0.05. n.s = not significant. β-actin was used as the protein loading control.

To determine if 6-shogaol treatment induces ROS generation via Nox4 expression, Nox4-specific siRNAs were transfected into A2780 and OVCAR-3 cells and then treated with 6-shogaol. The knockdown experiment of Nox4 suppressed the inhibition of cell viability and the increased LDH release, caspase-3 activity, ROS generation, and intracellular Ca^2+^ production in 6-shogaol-treated A2780 and OVCAR-3 cells compared to the control (Figure 7A–E). In Western blotting analyses, the loss of Nox4 downregulated Nox4, p-PERK, and CHOP levels in 6-shogaol-treated A2780 and OVCAR-3 cells compared to the control (Figure 7F). These results indicated that targeting Nox4 blocks ER stress and apoptotic cell death by inhibiting ROS generation in 6-shogaol-treated ovarian cancer cells. 

### 2.7. Gefitinib in Combination with 6-shogaol Overcomes Gefitinib Resistance by Inhibiting the EMT Phenomenon in Ovarian Cancer Cells

**Figure 8:**
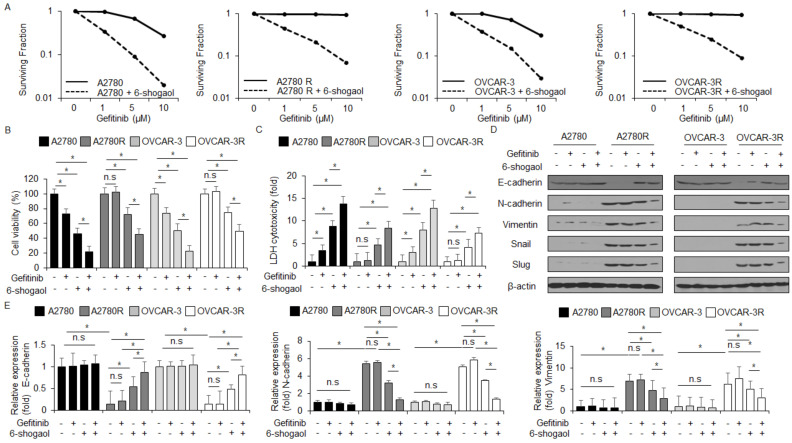
Gefitinib in combination with 6-shogaol overcomes resistance in gefitinib-resistant ovarian cancer cells. (**A**) A colony formation assay was performed at indicated doses (1, 5, or 10 μM) of gefitinib and then the survival fraction was calculated using the surviving fraction formula in A2780, A2780R, OVCAR-3, and OVCAR-3R; *, *p*<0.05. (**B**–**E**) A2780, A2780R, OVCAR-3, and OVCAR-3R cells were treated with 6-shogaol (30 μM, 24 h) and gefitinib (1 μM, 24 h). WST-1 assay and LDH assay were carried out along with a Western blot analysis for E-cadherin, N-cadherin, vimentin, Slug, and Snail and real-time RT-PCR for E-cadherin, N-cadherin, and vimentin; *, *p* < 0.05. n.s = not significant. β-actin was used as the RNA and protein loading control. β-actin was used as the protein loading control.

To identify if gefitinib/6-shogaol overcomes gefitinib resistance in ovarian cancer cells, gefitinib-resistant A2780R and OVCAR-3R cells were established by exposing gefitinib in A2780 and OVCAR-3 cells and then performing the colony formation assay, WST-1 assay, LDH assay, Western blot analysis, and real-time RT-PCR. The finding showed that 6-shogaol inhibits surviving fraction levels at indicated conditions (1, 5, and 10 µM) in A2780, A2780R, OVCAR-3, and OVCAR-3R cells when compared with control cells (Figure 8A). 6-shogaol inhibited cell viability and enhanced LDH release in A2780 and OVCAR-3 cells when compared with A2780R and OVCAR-3R cells, and gefitinib/6-shogaol mediated lower cell viability and higher LDH release in A2780 and OVCAR-3 cells when compared with A2780R and OVCAR-3R cells (Figure 8B,C). To determine if gefitinib/6-shogaol inhibits the EMT phenomenon in gefitinib-resistant ovarian cancer cells, we carried out Western blot analyses and real-time RT-PCR. In A2780 and OVCAR-3 cells, 6-shogaol, gefitinib, and gefitinib/6-shogaol showed no change in the expression levels of EMT-related markers, including E-cadherin, N-cadherin, vimentin, Snail, and Slug (Figure 8D,E and Supplementary Figure S1). However, E-cadherin was downregulated, and N-cadherin, vimentin, Snail, and Slug were upregulated in A2780R and OVCAR-3R cells compared to the control (Figure 8D,E). 6-shogaol and gefitinib/6-shogaol induced the upregulation of E-cadherin and the downregulation of N-cadherin, vimentin, Snail, and Slug in A2780R and OVCAR-3R cells compared to the control (Figure 8D,E). 

## 3. Discussion

A number of studies have reported that natural products play a potential role in the anti-cancer effect on cancer therapy [39,40]. High ROS generation regulates anti- or pro-oxidant defense systems in cancer cells and targeting oxidants may be a novel tumor therapeutic strategy [41,42]. In the present study, we studied the use of ginger bioactive compound 6-shogaol and suggested it could be a powerful anti-cancer agent in ovarian cancer. Furthermore, we investigated the detailed signaling pathway underlying apoptotic cell death in 6-shogaol-treated ovarian cancer cells. Our results herein identified that 6-shogaol induces ER stress and apoptotic cell death via Nox4 expression, ROS generation, and Ca^2+^ release in 6-shogaol-treated A2780 and OVCAR-3 cells. 6-shogaol mediates apoptotic cell death by activating the PERK-ATF4-CHOP axis. CHOP activated by 6-shogaol treatment binds to DR5 promoter, and it induces caspase-3-dependent apoptotic cell death. Gefitinib also induces apoptotic cell death via intracellular Ca^2+^ and ROS release and the ER stress pathway in glioma cells [43]. Moreover, 6-shogaol + gefitinib overcomes gefitinib resistance by inhibiting the EMT process and the activating ER stress in gefitinib-resistant ovarian cancer cells. 

Traditional herbal medicines and bioactive compounds exert a powerful anti-cancer effect with fewer side effects [44]. Furthermore, synergistic effects induced by bioactive compounds may suggest a novel anti-tumor therapeutic strategy in various chemo-resistant cancel cell lines [45]. Herbal medicine H3 + gemcitabine has anti-cancer effects such as G0/G1 cell cycle arrest, the inhibition of migration, and cytochrome C release in gemcitabine-resistant pancreatic cancer cells [46]. The basic ingredients of ginger are phenolic compounds, terpenes, polysaccharides, lipids, organic acids, and raw fiber [47]. Phenolic compounds, including shogaols and gingerols, possess various biological activities such as anti-cancer, anti-obesity, anti-oxidation, and anti-inflammation [48]. A number of reports have reported the potential roles of 6-shogaol in vitro and in vivo models. 6-shogaol exerts powerful anti-colorectal cancer effects via the anti-oxidant and anti-inflammatory effects in in vivo modes [49]. Moreover, 6-shogaol induces apoptosis by inhibiting the EMT phenomenon and the EGFR/PI3K/Akt pathway in oral squamous cell carcinoma [50]. Our results suggested that 6-shogaol exerts a potential anti-cancer effect via apoptotic cell death in ovarian cancer cells in vitro and in vivo. Furthermore, 6-shogaol induces ROS production via the activation of Nox4 and then mediates ER stress through intracellular Ca^2+^ generation. The results indicated that treatment with 6-shogaol has a ROS scavenging function, indicating that the anti-oxidant effect of 6-shogaol induced apoptotic cell death and ER stress in ovarian cancer.

Gefitinib (also named Iressa) is a potential anti-cancer drug for clinical cancer therapy, and it is known as an epidermal growth factor receptor (EGFR) tyrosine kinase inhibitor (TKI) [51]. EGFR is upregulated in 35~70% of epithelial ovarian cancers and the enhanced EGFR signaling pathway has been closely linked with the development of an invasive phenotype in various ovarian cancer cell types [52]. However, in various cancer types, gefitinib often induces chemo-resistance. Gefitinib in combination with DNA topoisomerase I inhibitor CPT-11 exerts a powerful anti-cancer effect via the activation of apoptosis and the inhibition of EGFR phosphorylation in vitro and in vivo [53]. Many reports have suggested that there is a powerful relationship between EMT and EGFR-TKI resistance [54]. EMT marker Twist knockdown induces caspase-3- and -7-dependent apoptotic cell death by inhibiting the phosphorylation of EGFR, Akt, vimentin, and Twist1, and by activating E-cadherin in EGFR-TKI gefitinib and AZD9291-treated gefitinib-resistant lung cancer cells [55]. We investigated the synergistic effect of 6-shogaol and gefitinib in gefitinib-resistant ovarian cancer cells, A2780R and OVCAR-3R. 6-shogaol + gefitinib overcame gefitinib resistance via the increase in E-cadherin and the inhibition of N-cadherin, vimentin, Slug, and Snail. Our finding indicates that gefitinib-resistant ovarian cancer cell lines acquired the EMT phenomenon. On the other hand, when we treated gefitinib-resistant ovarian cancer cell lines with 6-shogaol, it induced potential apoptotic cell death by inhibiting the EMT phenotype. In gefitinib-resistant in vitro models, many signaling pathways play potential roles for EMT induction. Therefore, more research is necessary to probe the detailed mechanism on the relationship between gefitinib resistance and EMT.

Although we identified the molecular pathway by 6-shogaol treatment in ovarian cancer cells and gefitinib-resistant ovarian cancer cells, and its intracellular ROS and Ca^2+^ production suppressed ovarian cancer cells and gefitinib-resistant ovarian cancer cell growth, how 6-shogaol-induced ER stress overcomes gefitinib resistance is still elusive. Similarly, the underlying mechanisms of 6-shogaol are yet unknown, though their anti-cancer effects are well reported. Increasing reports suggest that an excessive ER stress response is a novel therapeutic strategy to overcome EGFR-mediated resistance [56]. ER stress inducer tunicamycin induces apoptotic cell death and inhibits EGFR phosphorylation by suppressing the biosynthesis of N-linked oligosaccharides on the ER and Golgi [57,58]. Increasing evidence suggests that 6-shogaol treatment induces anti-cancer effects via blocking of the EGFR pathway, and EGFR-TKI gefitinib also exerts powerful anti-cancer effects by inhibiting the phosphorylation of EGFR and by activating the ER stress response. These results may suggest a novel therapeutic strategy in various cancer types and chemo-resistance models. The combination treatment with tunicamycin and EGFR-TKI erlotinib induces caspase-3 and PARP cleavage via the activation of pro-apoptotic and ER stress marker CHOP, and CHOP induction by combination treatment mediates apoptotic cell death by increasing death receptor 5 (DR5) [59,60]. Our results showed that 6-shogaol treatment induces apoptotic cell death and the ER stress response via intracellular Ca^2+^ generation, the phosphorylation of PERK and eIF2α, and the upregulation of GRP78, ATF4, DR5, and CHOP. In addition, 6-shogaol + gefitinib mediate the potential anti-cancer effect via apoptosis and ER stress in gefitinib-resistant ovarian cancer cells. Based on these findings, we suggest that 6-shogaol + gefitinib co-treatment may overcome EGFR-mediated resistance and induce a programmed cell death pathway via the excessive ER stress response in ovarian cancer cells and gefitinib-resistant cancer cells. 

**Figure 9:**
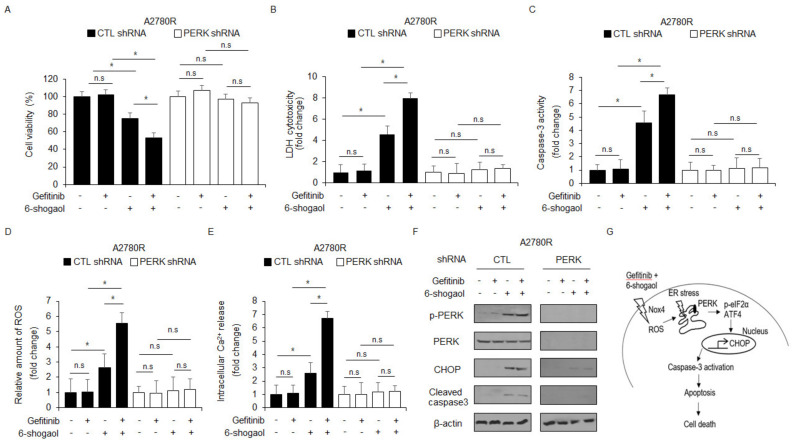
PERK knockdown blocks gefitinib/6-shogaol-mediated sensitivity in gefitinib-resistant A2780R cells. (**A**–**F**) After A2780R cells were transfected with PERK shRNA, PERK knockdown A2780R stable cell lines were established. These cells were treated with 6-shogaol (30 μM, 24 h) and gefitinib (1 μM, 24 h), and cell viability assays, LDH assays, caspase-3 activity assays, intracellular ROS assay, intracellular Ca^2+^ assays, and Western blot analysis were performed to examine p-PERK, PERK, CHOP, and cleaved caspase-3 expression; *, *p* < 0.05. n.s = not significant. β-actin was used as a protein loading control. (**G**) Schematic representation of ER stress and apoptotic cell death signaling pathways induced by gefitinib plus 6-shogaol in ovarian cancer and gefitinib-resistant ovarian cancer cells.

In conclusion, we studied the ginger bioactive compound 6-shogaol and identified the anti-cancer effect of 6-shogaol in vitro and in vivo in ovarian cancer. 6-shogaol induces cell death and ER stress-related markers, such as GRP78, p-PERK, p-eIF2α, ATF4, and CHOP, via the upregulation of Nox4, and ROS and intracellular Ca^2+^ release in ovarian cancer cells. Moreover, we established gefitinib-resistant ovarian cancer cells, A2780R and OVCAR-3R from the gefitinib-sensitive human ovarian cancer cell lines A2780 and OVCAR-3, and combined treatment with gefitinib and 6-shogaol overcomes gefitinib resistance via the activation of ER stress and the suppression of EMT processes such as the inhibition of E-cadherin and the increase in N-cadherin, vimentin, Slug, and Snail (Figure 9G).

### PERK Inhibition Knockdown Blocks gefitinib/6-shogaol-Induced Cell Death and ER Stress in Ovarian Cancer Cells

To determine if gefitinib/6-shogaol can overcome gefitinib-mediated resistance, we established PERK knockdown stable cells using PERK shRNA in A2780R cells. PERK knockdown stable cells were treated with 6-shogaol, gefitinib, or gefitinib/6-shogaol and cell viability assays, LDH assays, caspase-3 activity assays, intracellular ROS assays, intracellular Ca^2+^ assays, and Western blot analyses were performed. In A2780R stable cells, 6-shogaol inhibited cell viability and enhanced LDH generation. Gefitinib/6-shogaol induced even lower cell viability, higher LDH production, caspase-3 activity, ROS release, and Ca^2+^ generation, but gefitinib alone had no effect. In contrast, in PERK knockdown stable A2780R cells, 6-shogaol, gefitinib, or gefitinib/6-shogaol had no effect (Figure 9A–E). Western blot analysis indicated that 6-shogaol alone and gefitinib/6-shogaol induced the expression of p-PERK, CHOP, and caspase-3 cleavage, but gefitinib alone had no effect (Figure 9F). Furthermore, in PERK knockdown stable A2780R cells, 6-shogaol, gefitinib, and gefitinib/6-shogaol had no effect on the expression of p-PERK, CHOP, and caspase-3 cleavage.

## 4. Materials and Methods

### 4.1. Reagents

Compounds were obtained as follows: diphenyleneiodonium (Nox or ROS inhibitor, DPI, Sigma Aldrich, St Louis, MO, USA), N-acetylcysteine (a ROS inhibitor, NAC, Sigma Aldrich, St Louis, MO, USA), Z-VAD-FMK (caspase inhibitor, Sigma Aldrich, St Louis, MO, USA), 6-shogaol (Sigma Aldrich, St Louis, MO, USA), and thapsigargin (ER stress inducer, TG, Millipore, Bedford, MA, USA).

### 4.2. Cell Culture

A2780, OVCAR-3, Caov-3, and SK-OV-3 human ovarian cancer cell lines were obtained from the Korean Cell Line Bank (Cancer Research Center, Seoul National University, Seoul, Korea). These cells were cultured in DMEM (Gibco, CA, USA) supplemented with 10% fetal bovine serum (FBS), 100 U/mL penicillin, and 100 mg/mL streptomycin (all from Gibco, CA, USA) at 37 °C under a humidified 95/5% (*v*/*v*) mixture of air and CO_2_ atmosphere incubator.

### 4.3. Cell Viability

A2780, OVCAR-3, Caov-3, and SK-OV-3 human ovarian cancer cell lines were seeded into a 96-well plate with DMEM medium and grown for 24 h, and then cells were treated with 6-shogaol for 24 h. Cell viability was performed using a WST-1 assay (Roche, CA, USA) according to the manufacturer’s protocols. Cell absorbance was measured at 450 nm using an enzyme-linked immunosorbent assay reader (SpectraMax190, Microplate Reader, Molecular Devices, CA, USA).

### 4.4. LDH Assay

A2780, OVCAR-3, Caov-3, and SK-OV-3 human ovarian cancer cell lines were seeded into a 96-well plate with a growth medium and grown for 24 h, and then cells were treated with 6-shogaol for 24 h. LDH cytotoxicity assay (LDH cytotoxicity assay) was performed according to the manufacturer’s protocols. The fluorescence was determined by measuring the absorbance of the samples at 490 or 492 nm using the ELISA reader (SpectraMax190, Microplate Reader, Molecular Devices, CA, USA).

### 4.5. Caspase-3 Activity Assays 

A2780 and OVCAR-3 human ovarian cancer cell lines were seeded into a 6-well plate with a growth medium and grown for 24 h. Caspase-3 activity assays (the Biovision colorimetric caspase-3 assay kit) were performed according to the manufacturer’s protocols. The fluorescence was analyzed at 405 nm using a spectrophotometer (Molecular Devices, CA, USA). 

### 4.6. Intracellular Ca^2+^ Assays

A2780 and OVCAR-3 human ovarian cancer cell lines were seeded into a 96-well plate with a growth medium, Then, the cells were treated with 6-shogaol. Intracellular calcium assays were performed using a Ca^2+^ Assay Kit (Colorimetric) (Abcam; ab102505) as described in the supplier’s manual. The fluorescence was measured and analyzed by the absorbance of the samples at 575 nm using a microplate reader (Molecular Devices, CA, USA).

### 4.7. Intracellular ROS Assays

A2780 and OVCAR-3 human ovarian cancer cell lines were seeded and incubated into a 96-well plate with a growth medium, and then cells were treated with 6-shogaol. After these treatments, cells were incubated with the cell-permeant 2′7′-dichlorodihydrofluorescein diacetate (CM-H_2_DCFDA, Invitrogen) for 30 min at 37 °C as described in the supplier’s protocol. The fluorescence was measured and analyzed by the absorbance of the samples at 495 (Ex)/525 (Em) nm using a microplate reader (Molecular Devices, CA, USA).

### 4.8. Establishment of Gefitinib-Resistant A2780 and OVCAR-3 Cell Lines

A2780 and OVCAR-3 human ovarian cancer cell lines were seeded into 60 mm dishes and exposed to increasing doses for 6 months. For the establishment of gefitinib-resistant A2780R and OVCAR-3R cell lines, gefitinib (30 μΜ) treatment was started and was gradually enhanced to 60 nM over a period of 6 months. 

### 4.9. Colony Formation Assay

Human ovarian cancer cells and gefitinib-resistant human ovarian cancer cells (A2780, A2780R, OVCAR-3, and OVCAR-3R) were seeded onto 60 mm dishes with a growth medium and grown for 24 h at 37 °C CO_2_ incubators. Cells were incubated for 10 days to colony formation and then the colonies were stained with 0.5% crystal violet (Amresco, Solon, OH, United States). To calculate the survival fraction, the number of colonies formed were divided by the number of seeded cells formed of the control plate.

### 4.10. Transfection

A2780 and OVCAR-3 human ovarian cancer cell lines in a 6-well plate were transfected with double-stranded siRNAs (30 nmol/mL) such as GRP78 (Santacruz), PERK (Santacruz), CHOP (Bioneer), DR5 (Santacruz), and Nox4 (Santacruz), for 24 h using Lipofectamine 2000 reagent (Invitrogen) according to the manufacturer’s protocol. 

### 4.11. Isolation of Total RNA and Protein

Total RNA from human ovarian cancer cell lines A2780 and OVCAR-3 in a 100 mm cell culture dish was prepared using Trizol reagent according to the manufacturer’s instructions (Invitrogen). The protein of cell lysates was collected by radioimmunoprecipitation assay (RIPA) lysis buffer (Bio-Rad). The supernatant was analyzed to quantify protein using the BCA method (Thermo Scientific, CA, USA). 

### 4.12. Real-Time PCR and Western Blot Analyses

Real-time RT PCR performed in triplicate using an ABI Power SYBR Green PCR Master Mix (Applied Biosystems) with E-cadherin-specific primers (5′- GAACGCATTGCCACATACAC-3′ (sense) and 5′-GAATTCGGGCTTGTTGTCAT-3′ (antisense)), N-cadherin-specific primers (5′-GGCATACACCATG CCATCTT-3′ (sense) and 5′-GTGCATGAAGGACAGCCTCT-3′ (antisense)), vimentin-specific primers (5′-GAGAACTTTGCCGTTGAAGC-3′ (sense) and 5′-GCTTCCTGTAGGTGGCAATC-3′ (antisense)), ATF4-specific primers ((5′-AAGCCTAGGTCTCTTAGATG-3′ (sense) and 5′- TTCCAGGTCATCTATACCCA-3′ (antisense)), and CHOP-specific primers ((5′- ATGAGGACCTGCAAGAGGTCC-3′ (sense) and 5′-TCCTCCTCAGTCAGCCAAGC-3′ (antisense)) on a Roche LightCycler 96 System (Roche). RNA quantities were normalized with β-actin primers (5′-AAGGCCAAC CGCGAGAAGAT-3′ (sense) and 5′-TGATGACCTGGCCGTCAGG-3′ (antisense)), and gene expression analyses were quantified according to the 2^-ΔCt^ method. In Western blot analysis, cells were solubilized in RIPA lysis buffer (Bio-Rad). Then, blocked membranes were incubated overnight at 4 °C with primary antibodies. The primary antibodies used included β-actin (Santa Cruz, 1:1000, sc-47778), eIF2α (Santa Cruz, 1:1000, sc-133132), GRP78 (Santa Cruz, 1:1000, sc-166490); CD63 (Abcam, 1:1000, ab216130); Nox4 (Proteintech, 1:1000, 14347-1-AP); and cleaved caspase-3 (Cell Signaling, 1:1000, #9664), cleaved caspase-8 (Cell Signaling, 1:1000, #9748), cleaved caspase-9 (Cell Signaling, 1:1000, #20750), p-PERK(Thr980) (Cell Signaling, 1:1000, #3179), PERK (Cell Signaling, 1:1000, #5683), p-eIF2α (Ser51) (Cell Signaling, 1:1000, #3398), ATF4 (Cell Signaling, 1:1000, #11815), CHOP (Cell Signaling, 1:1000, #2895), DR4 (Cell Signaling, 1:1000, #42533), DR5 (Cell Signaling, 1:1000, #8074), E-cadherin (Cell Signaling, 1:1000, #14472), N-cadherin (Cell Signaling, 1:1000, #13116), Slug (Cell Signaling, 1:1000, #9585), Snail (Cell Signaling, 1:1000, #3879), and vimentin (CellSignaling, 1:1000, #5741). After membrane washing, it was incubated for 40 min at room temperature with a 1:4000 dilution of HRP-conjugated secondary antibodies. The secondary antibodies used included anti-mouse anti-rabbit IgG HRP-linked antibody (Santa Cruz, sc-2357) and m-IgGK BP-HRP-linked antibody (Santa Cruz, sc-516102). The membranes were analyzed by ECL Prime Western Blotting Detection Reagents (AmerSham, UK). 

### 4.13. Exosome Isolation

A2780 and OVCAR-3 human ovarian cancer cell lines were treated with 6-shogaol at the dose shown and then exosomes were obtained from the supernatant of 6-shogaol-treated A2780 and OVCAR-3 cells according to the manufacturer’s protocol (Total Exosome Isolation Reagent (for cell culture media), Thermo Fisher Scientific). Protein concentration was measured using the BCA method (Thermo Scientific). These protein loading samples (10 μg) were also quantified by Ponceau S staining and were subjected to Western blotting. Positive exosomes were identified using the exosome marker CD63.

### 4.14. Animals

For the animal study, five-week-old, female, athymic BALB/c nude mice (*nu/nu*) were purchased from OrientBio, Inc. (Daejeon, Korea), and maintained for 1 week with free access to sterile standard mouse chow (NIH-7 open formula) and water before use. Mice were housed randomly at 50 ± 20% humidity and approximately 21 ± 2 °C on a 12 h light–dark cycle (*n* = 10 mice/group). All animal experimental procedures were performed according to the National Institutes of Health guidelines and a protocol approved by the Institutional Animal Care and Use Committee of Kyung Hee University.

### 4.15. Tumor xenograft Mouse Models

For the mice xenograft experiment, mice, aged six weeks, were inoculated with an A2780 human ovarian cancer cell line by subcutaneously (sc) implanting 1 × 10^7^ cultured cells into the right thigh. Six days later, mice were grouped randomly (n = 10 per group) and 6-shogaol (40 or 60 mg/kg) was administered intraperitoneally (ip) once a day for two days. Tumor sizes on two axes (*L,* longest axis; *W,* shortest axis) were measured three times per week using Vernier calipers. Tumor volume was calculated as (*L* × *W*^2^)/2 (mm^3^).

### 4.16. Chromatin Immunoprecipitation (ChIP) Assay

To identify if 6-shogaol induces the binding of CHOP on the DR5 promoter, ChIP assays were performed using EZ ChIP Chromatin Immunoprecipitation Kit (Millipore, Billerica, MA, USA) as described in the supplier’s protocol. Real-time PCR primers (5′-AGGTTAGTTCCGGTCCCTTC-3′ (sense) and 5′- CAACTGCAAATTCCACCACA-3′ (antisense)) were designed to amplify the CHOP binding site at the DR5 gene promoter. 

### 4.17. Statistical Analysis

All results were confirmed in at least three independent experiments; Student’s *t*-test was used to compare the means of quantitative data between groups, and a *p-*value <0.05 was considered statistically significant.

## Data Availability

Not applicable.

## Supplementary Materials

The following supporting information can be downloaded at: https://www.mdpi.com/article/10.3390/ijms24032639/s1, Figure S1: The intensity of Western blot analysis for E-cadherin, N-cadherin, vimentin, Slug, and Snail.
