# Supplementary material for: 6-Shogaol Overcomes Gefitinib Resistance via ER Stress in Ovarian Cancer Cells
Source: Int J Mol Sci. 2023 Jan 30;24(3):2639. doi: 10.3390/ijms24032639 (PMC9916959; doi:10.3390/ijms24032639)

Western blotting whole blot:

Tae Woo Kim

Figure S1. Whole blots for Figure 1H

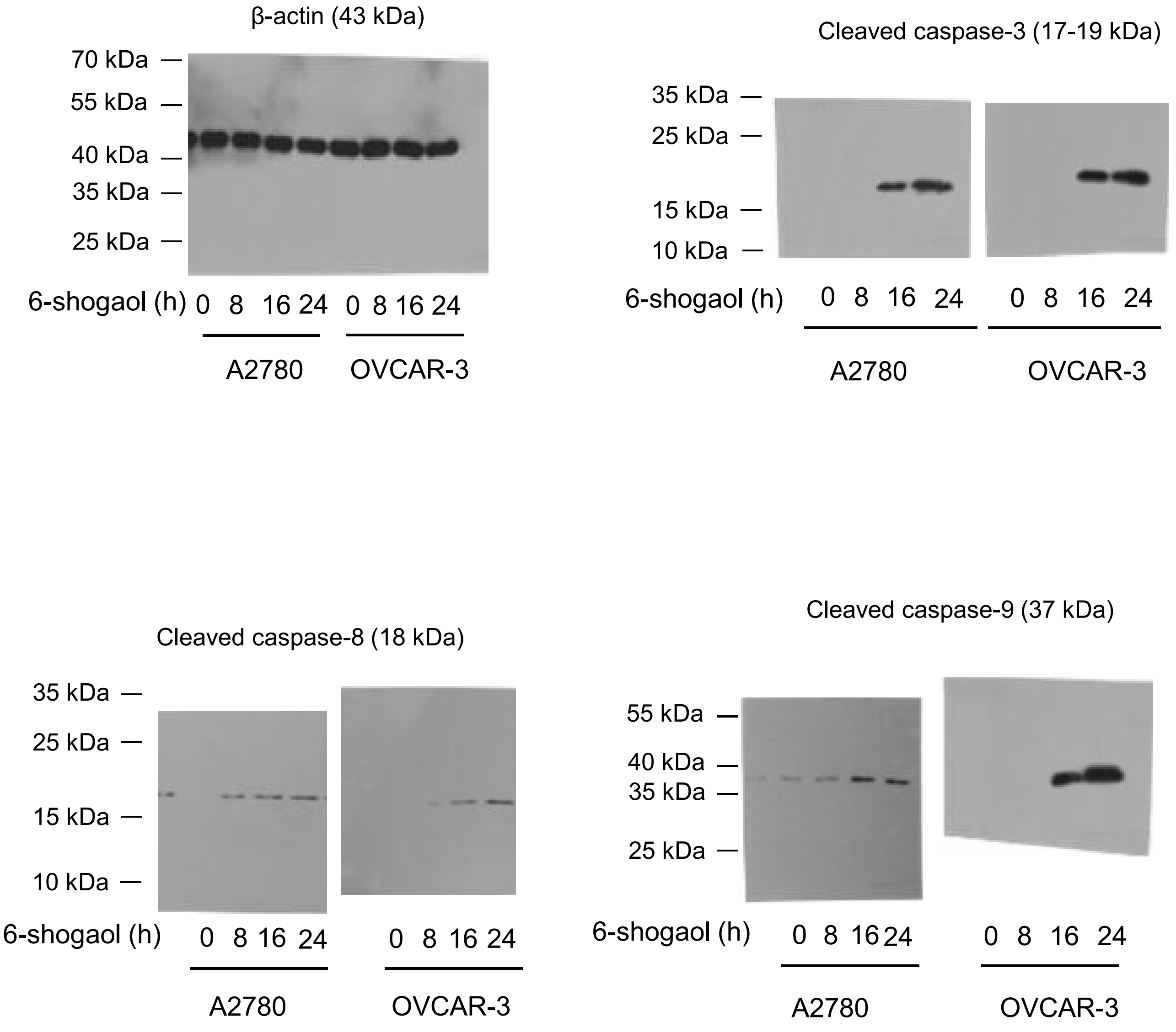

Whole blots for Figure 1L

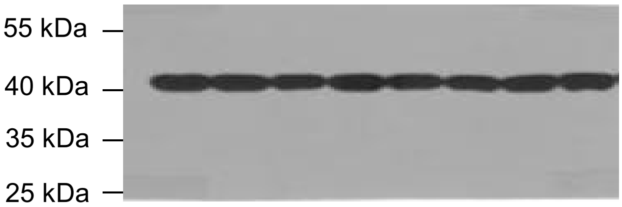

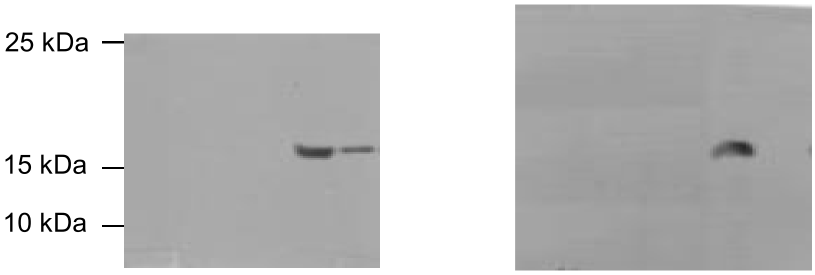

Whole blots for Figure 2C

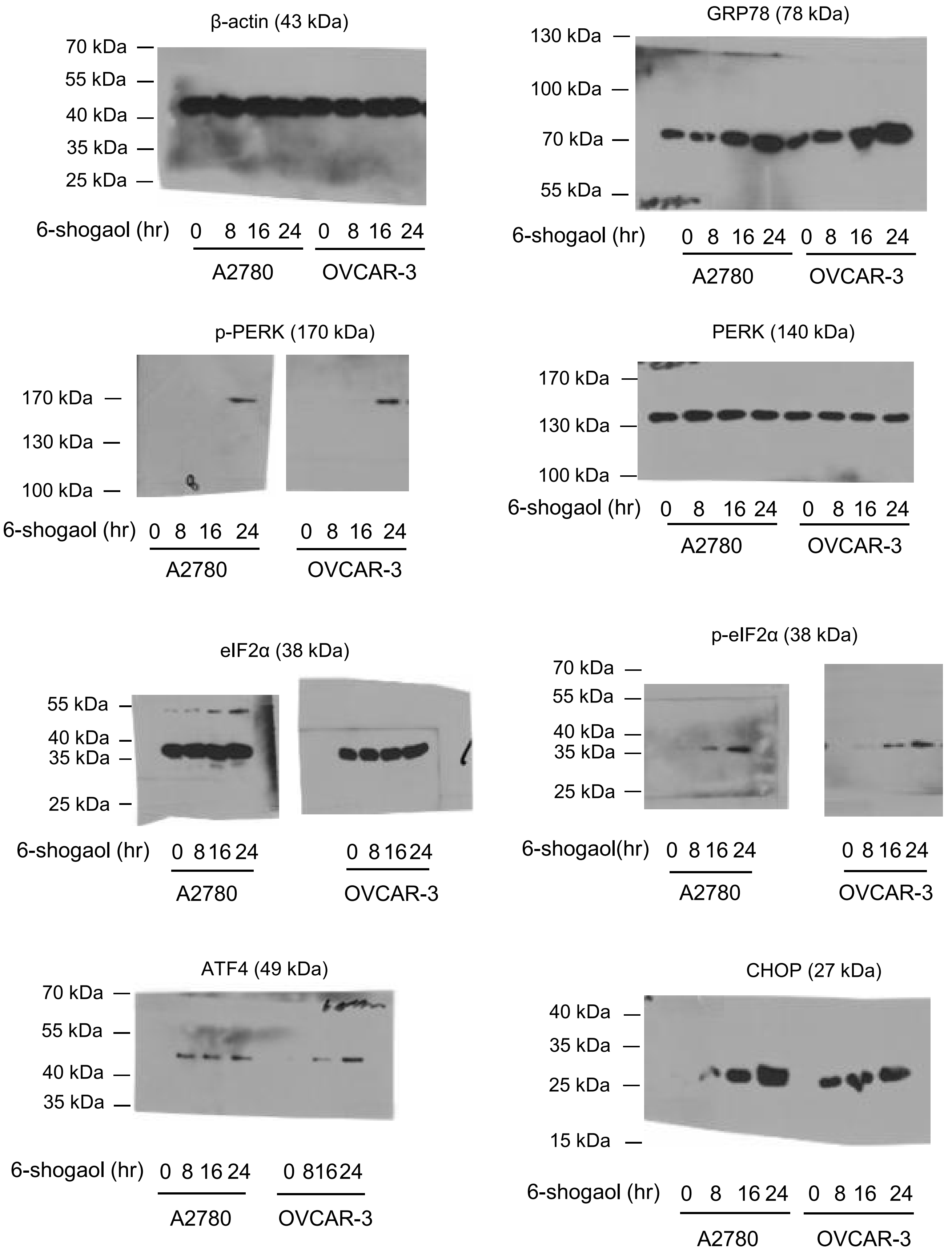

Whole blots for Figure 2D

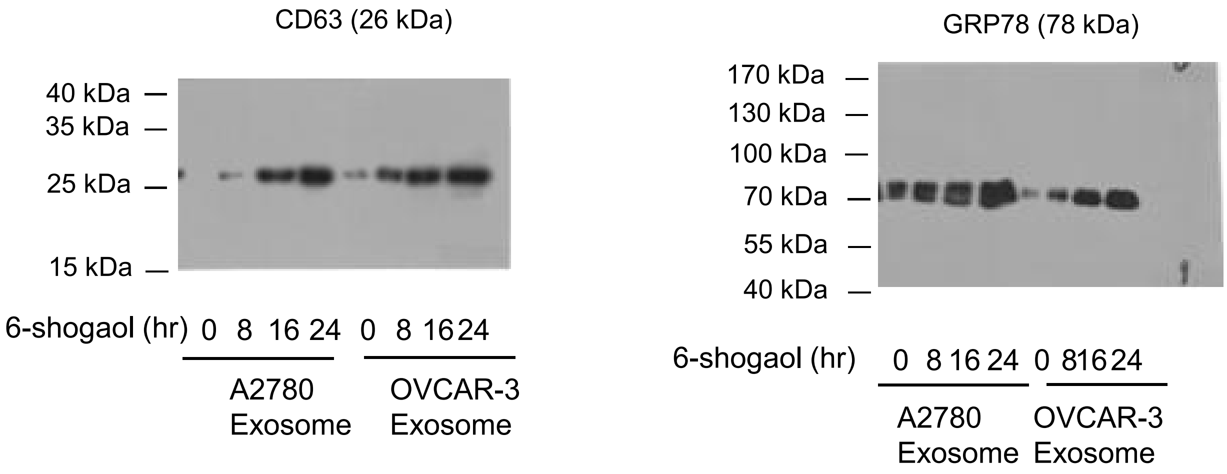

Whole blots for Figure 2H

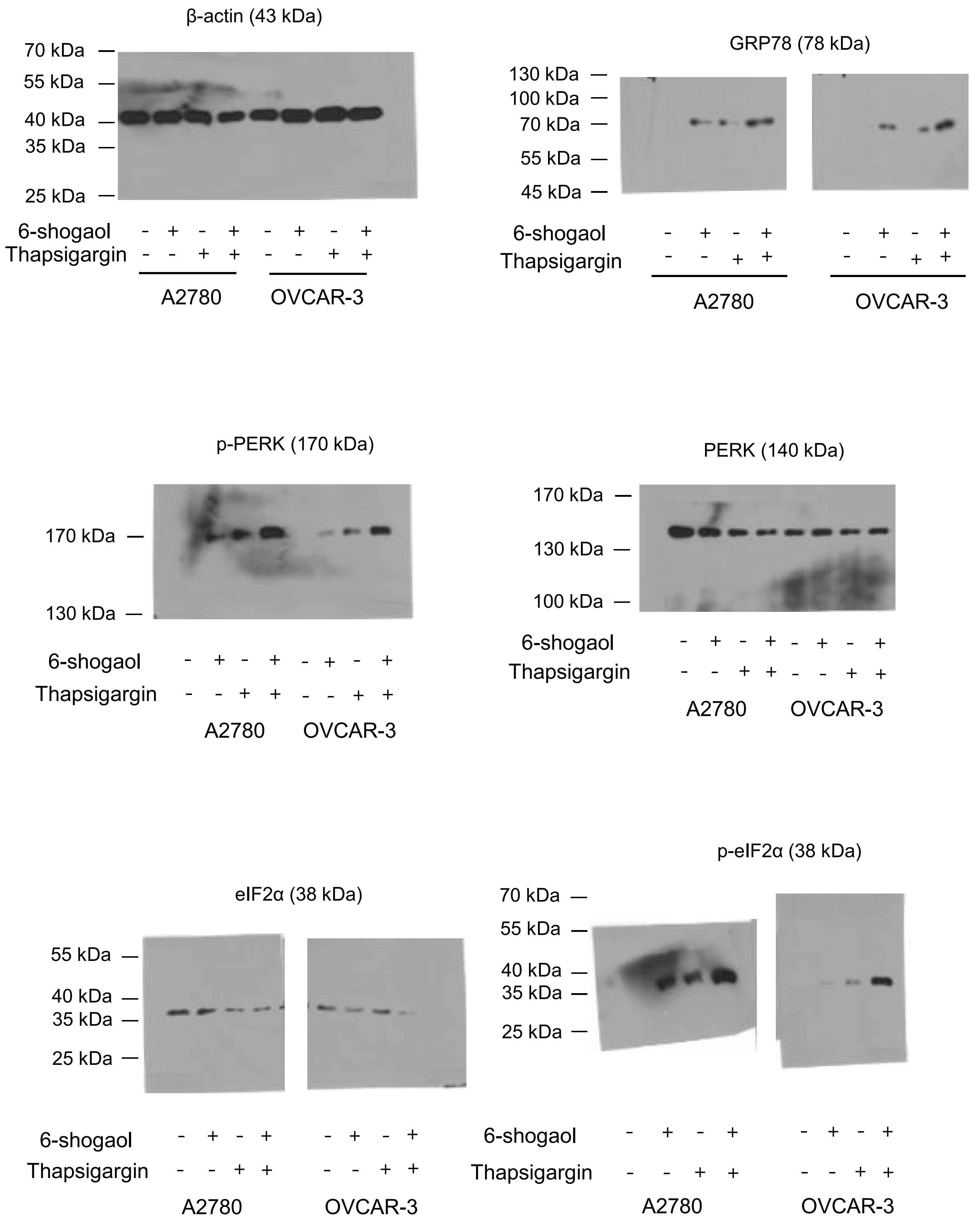

Whole blots for Figure 2H

CHOP (27 kDa)

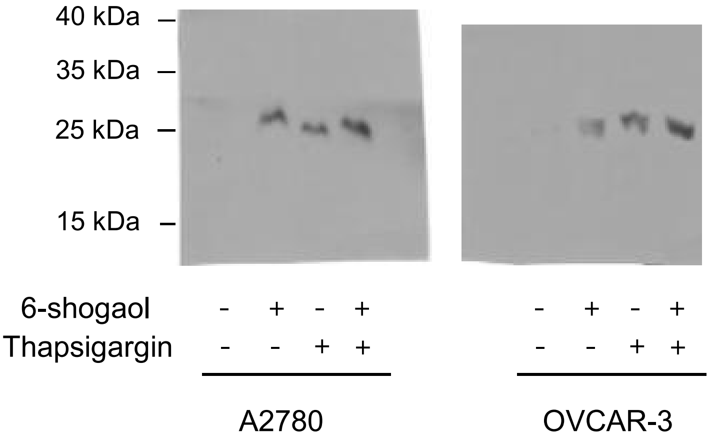

Cleaved caspase-3 (17-19 kDa)

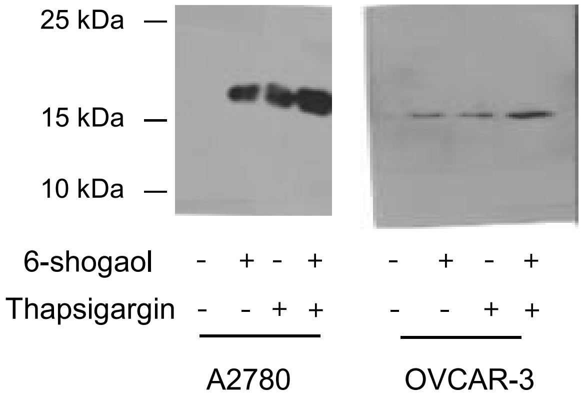

Whole blots for Figure 3D

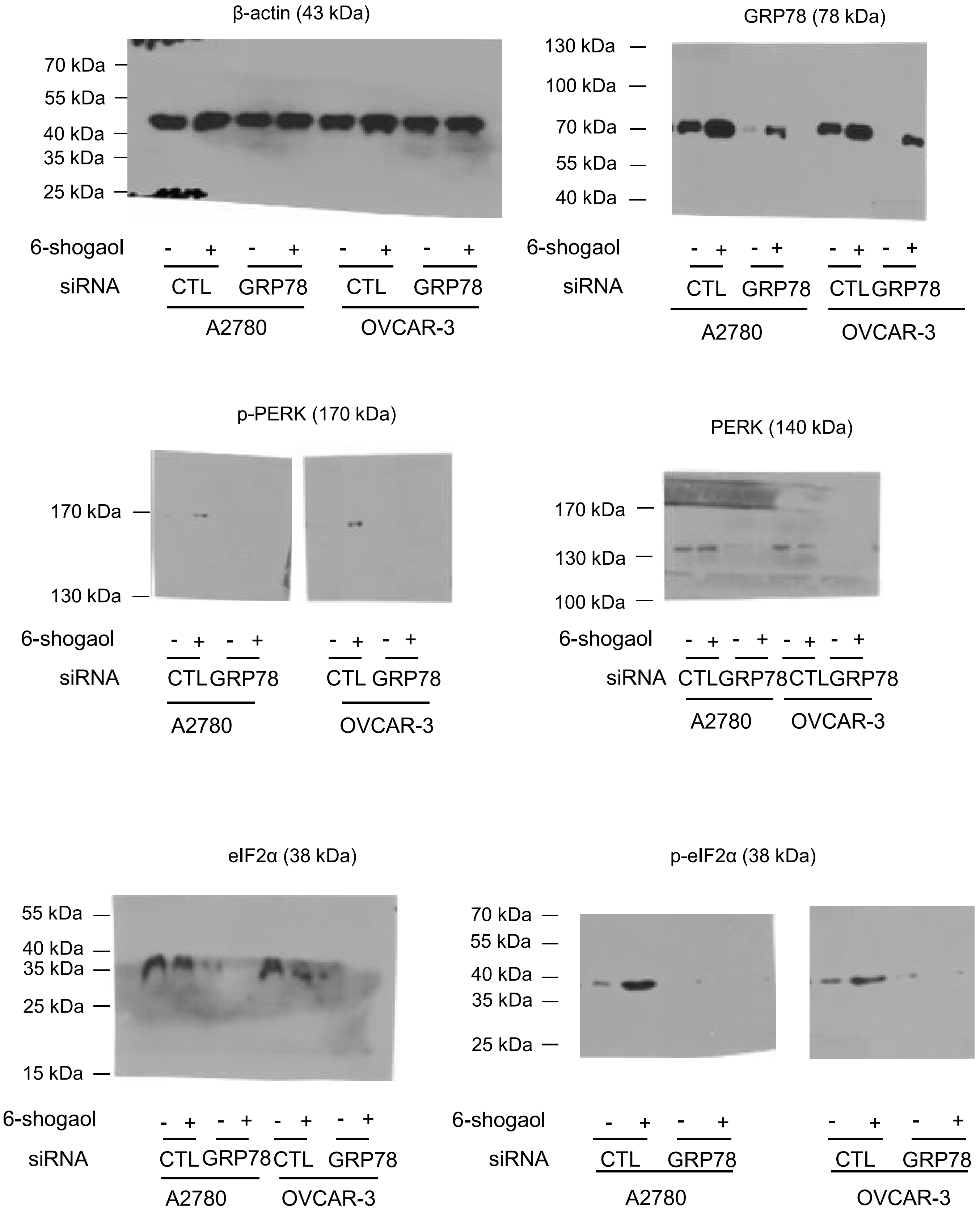

Whole blots for Figure 3D

CHOP (27 kDa)

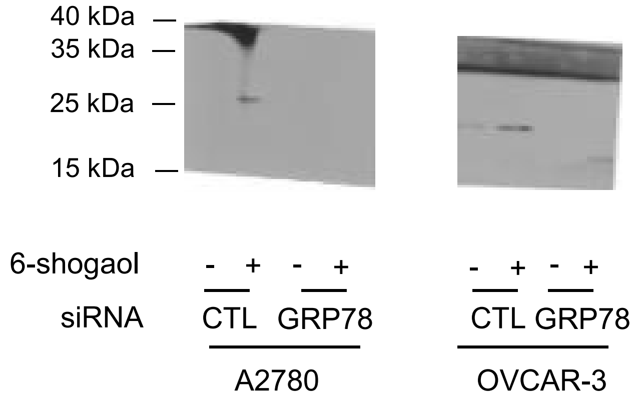

Cleaved caspase-3 (17-19 kDa)

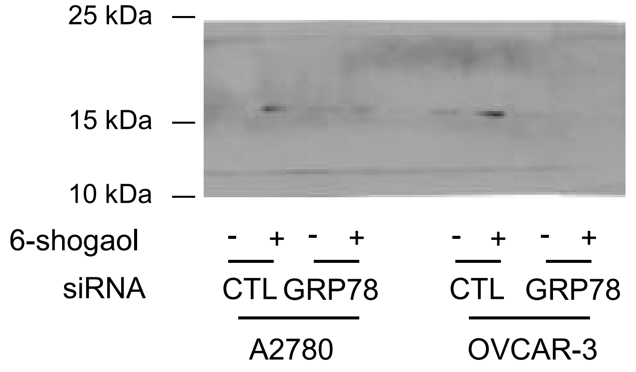

Whole blots for Figure 3H

β-actin (43 kDa)

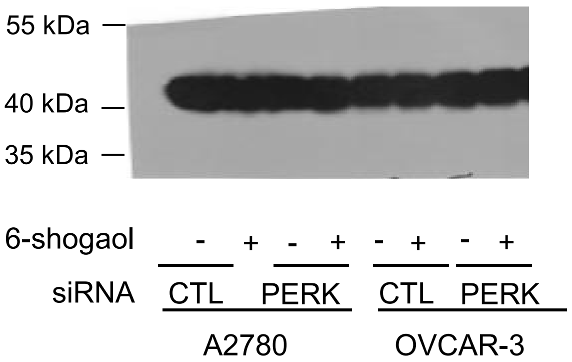

Cleaved caspase-3 (17-19 kDa)

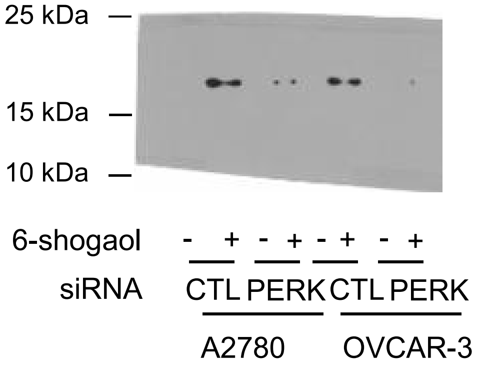

p-PERK (170 kDa)

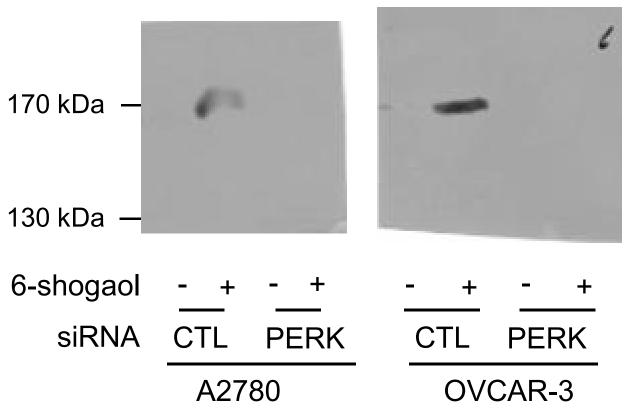

PERK (140 kDa)

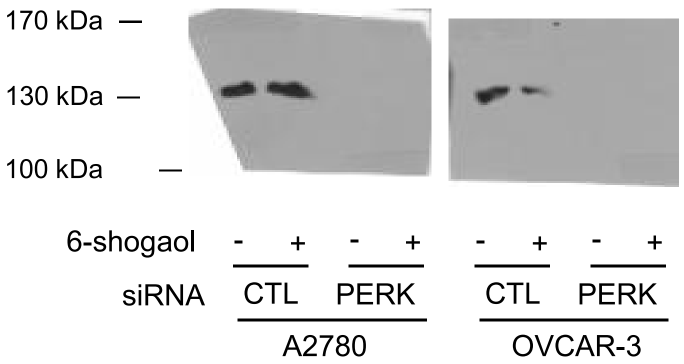

eIF2α (38 kDa)

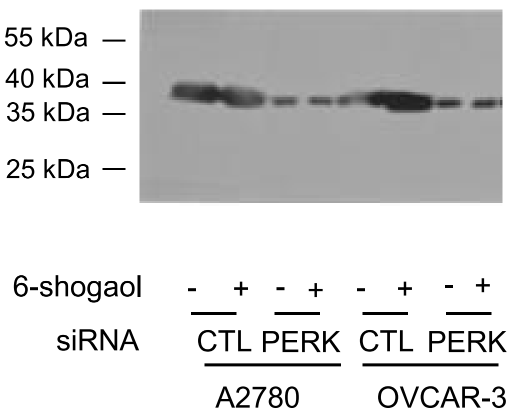

p-eIF2α (38 kDa)

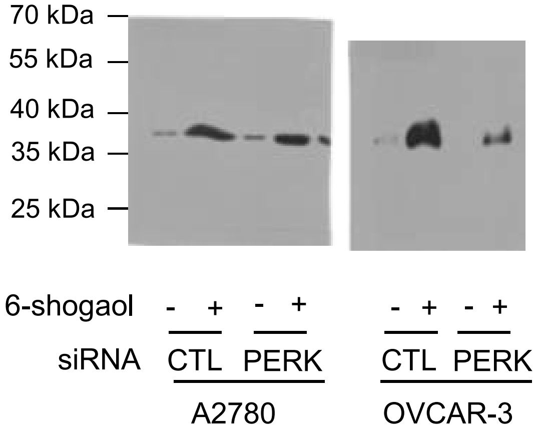

Whole blots for Figure 3H

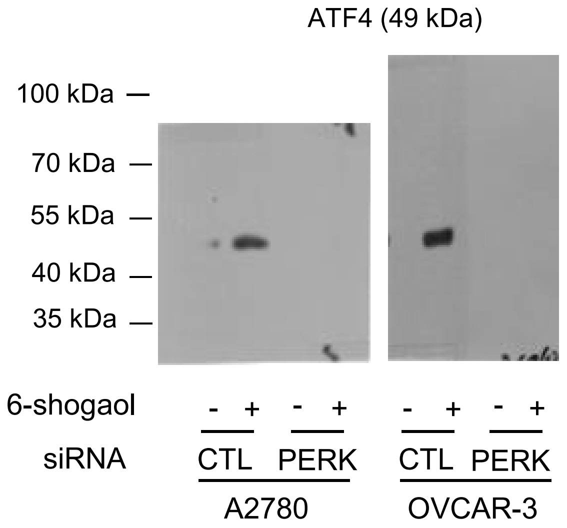

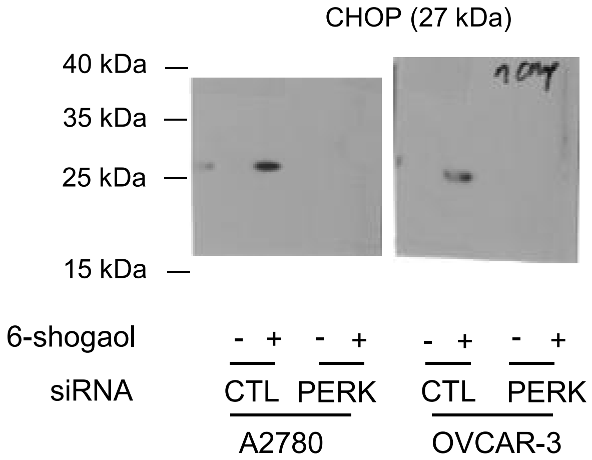

Whole blots for Figure 4C

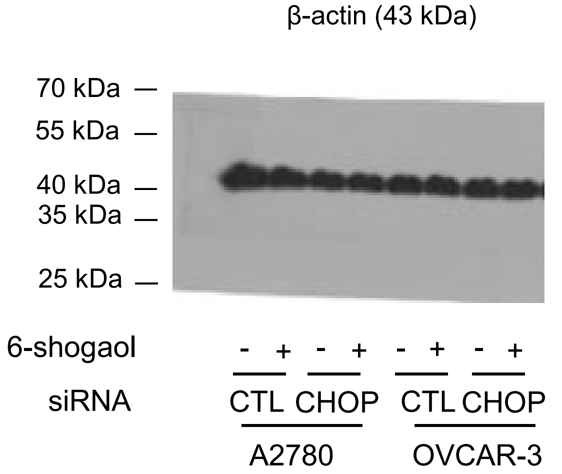

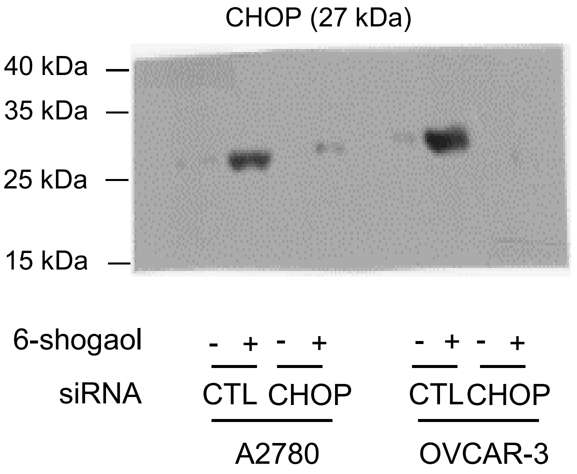

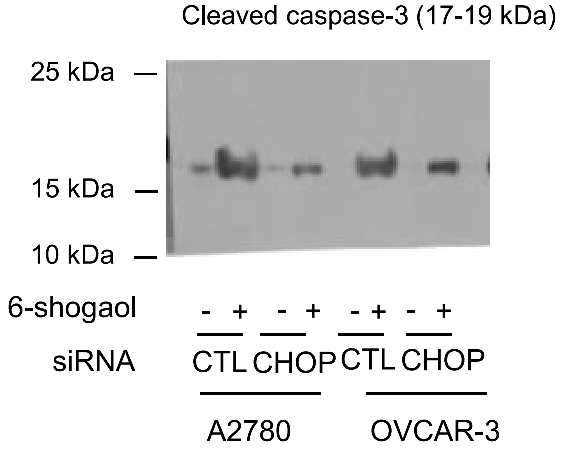

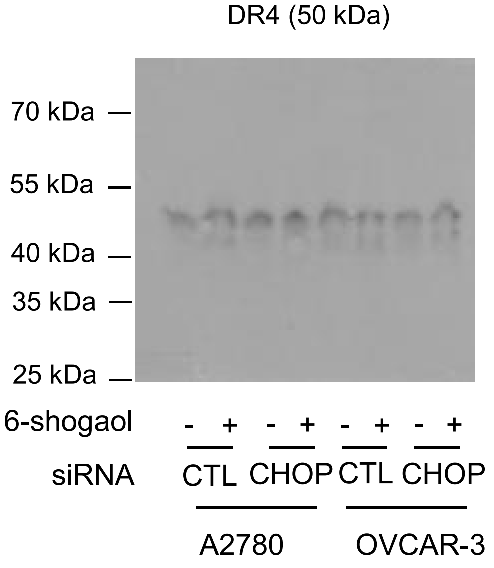

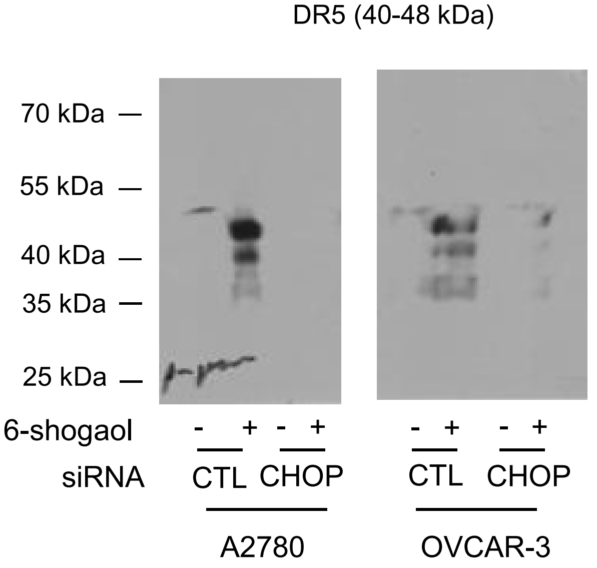

Whole blots for Figure 4H

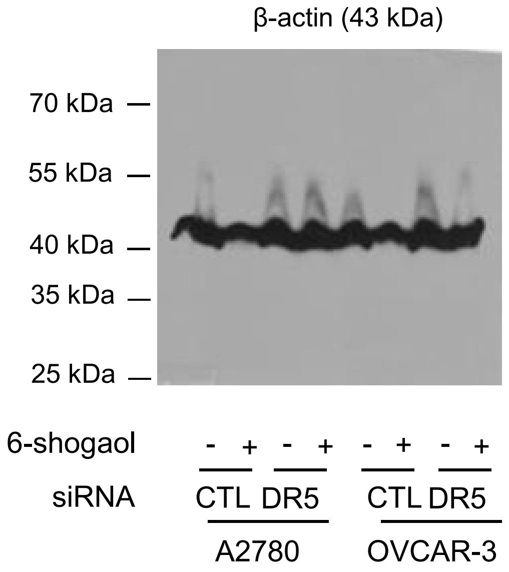

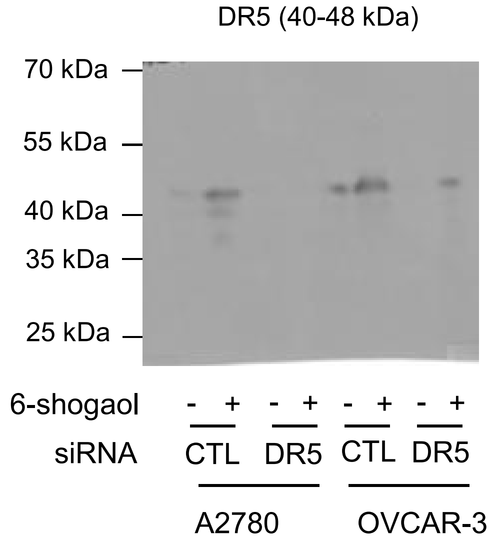

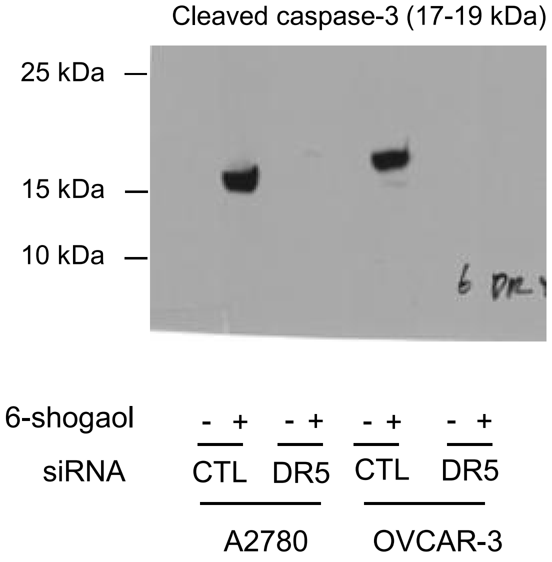

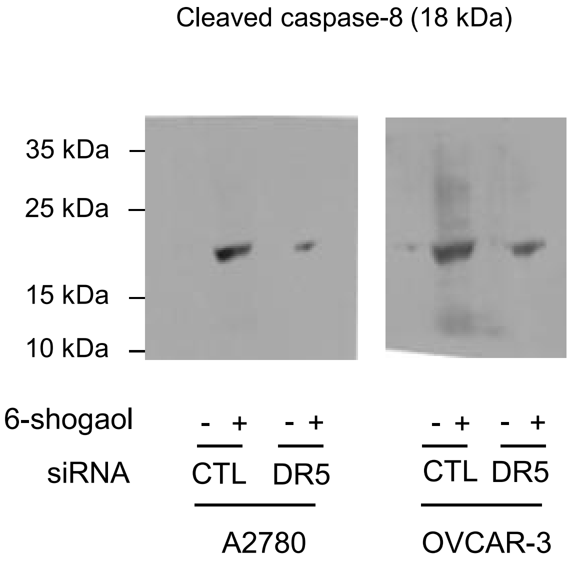

Whole blots for Figure 5G

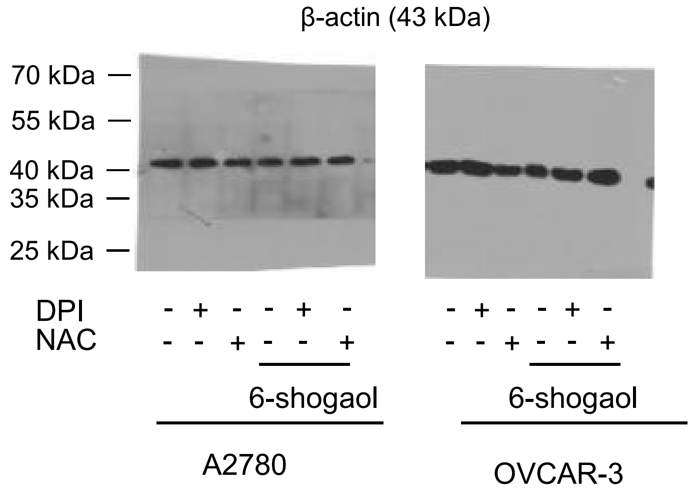

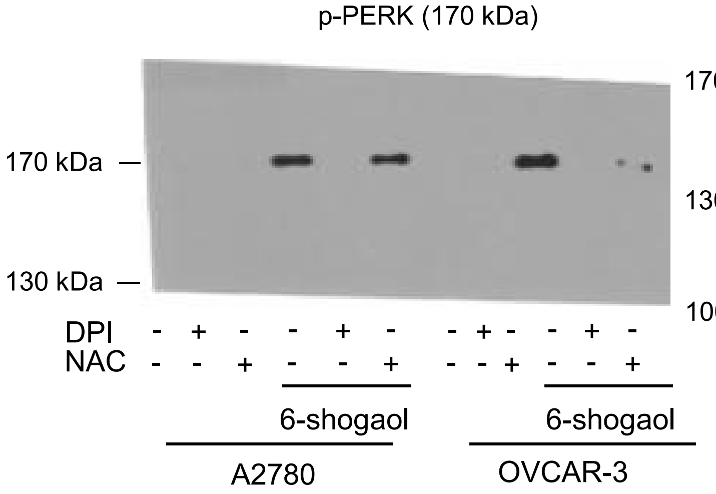

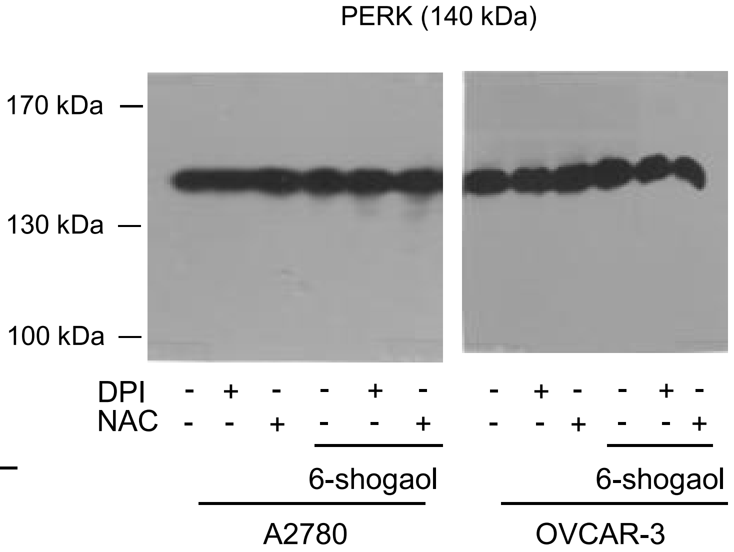

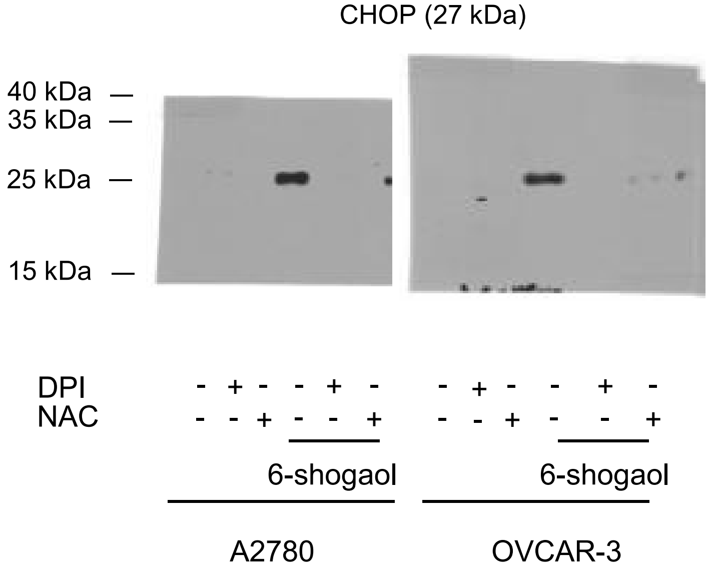

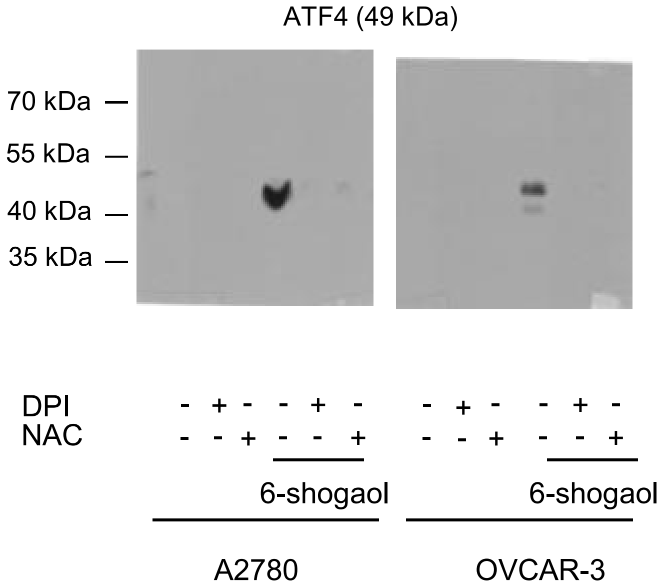

Whole blots for Figure 6F

β-actin (43 kDa)

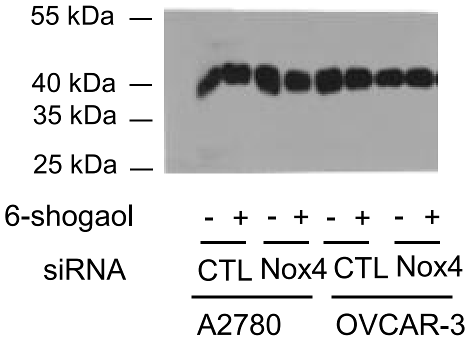

CHOP (27 kDa)

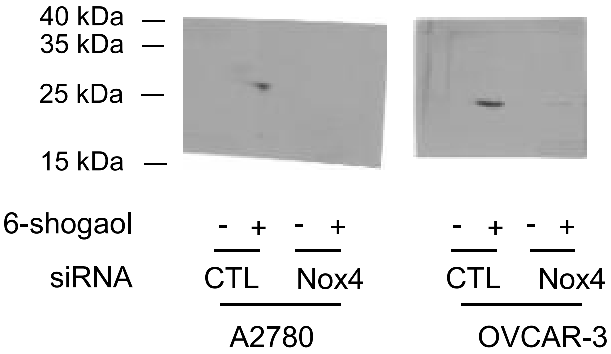

Nox4 (62 kDa)

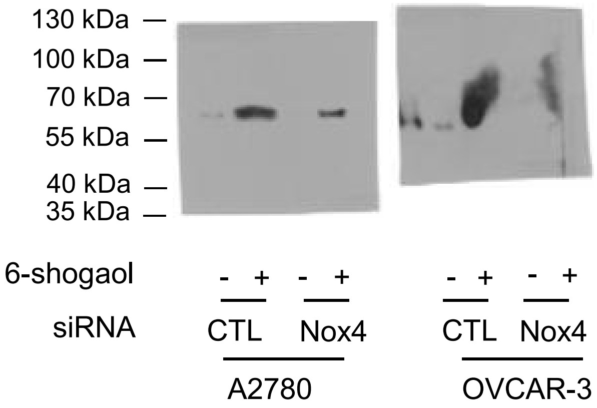

PERK (140 kDa)

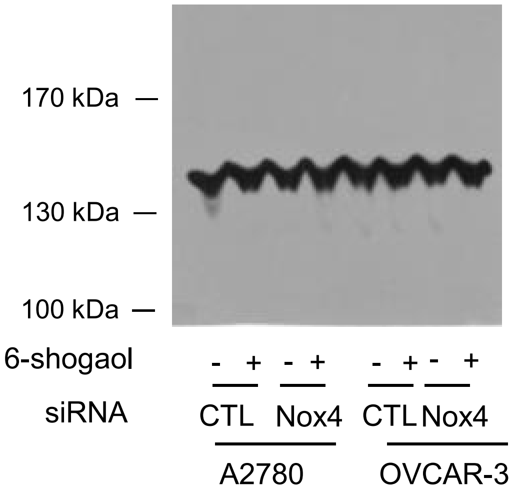

p-PERK (170 kDa)

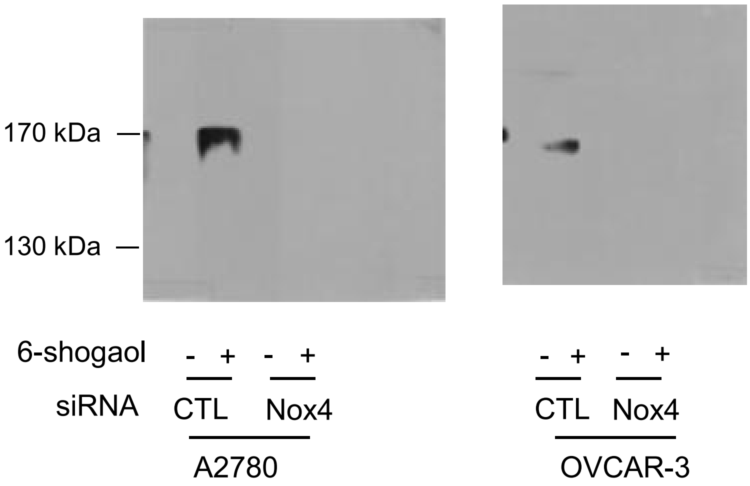

Whole blots for Figure 7D

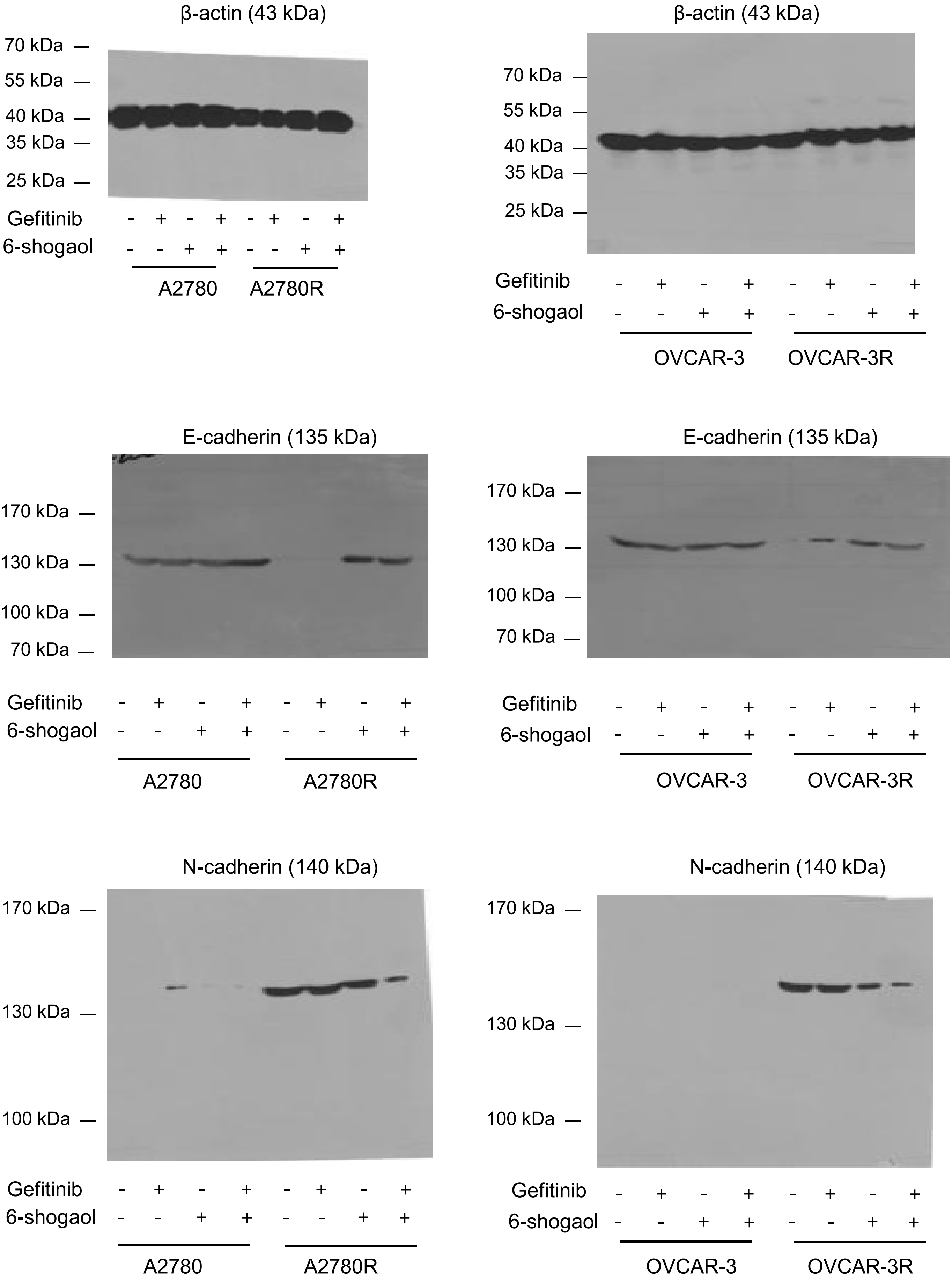

Whole blots for Figure 7D

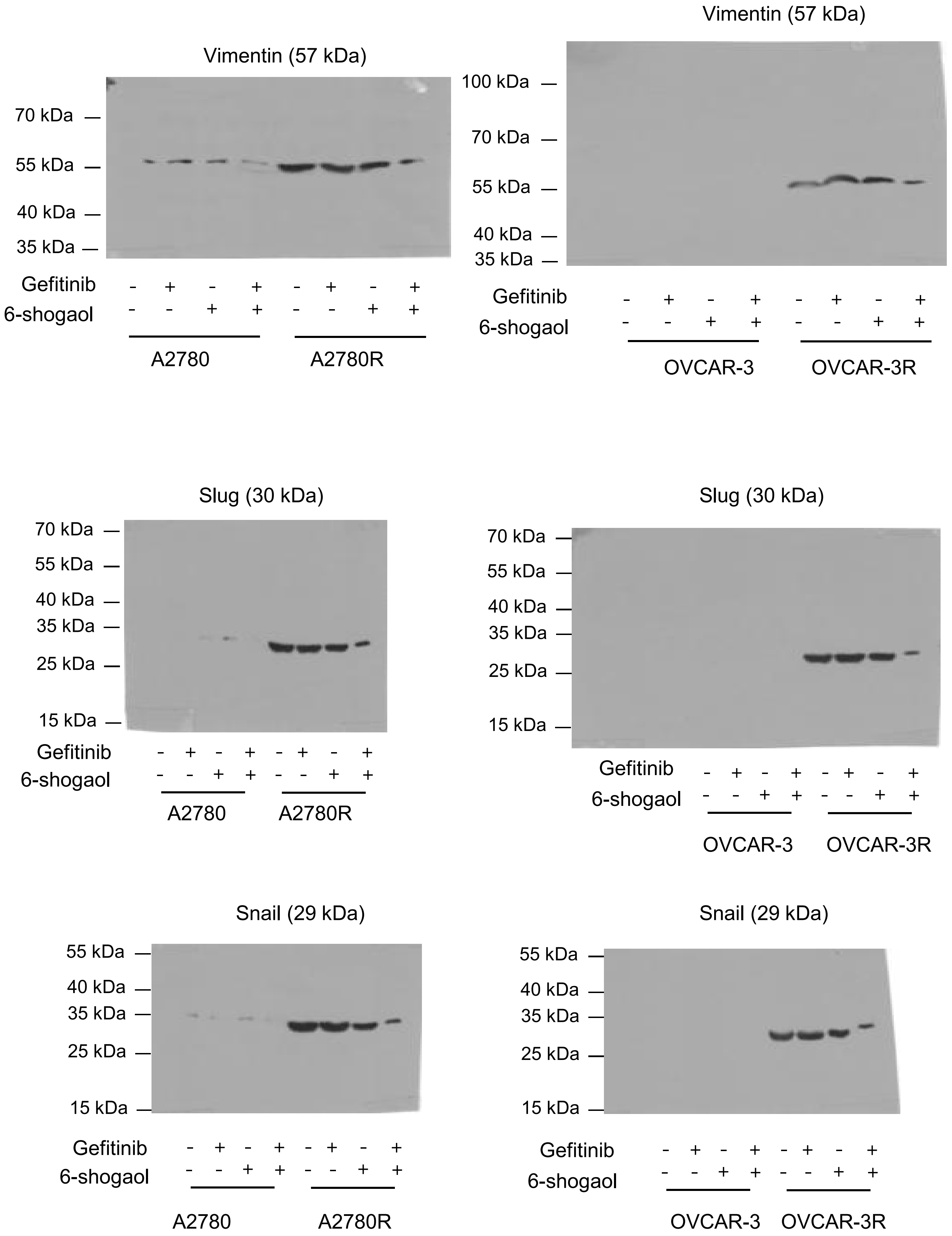

Whole blots for Figure 8F

β-actin (43 kDa)

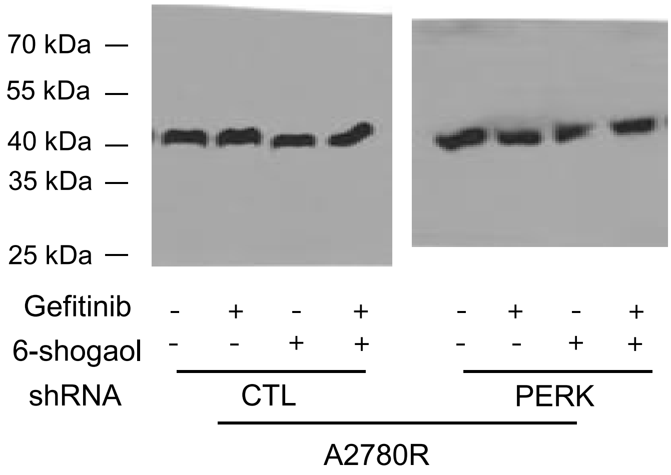

p-PERK (170 kDa)

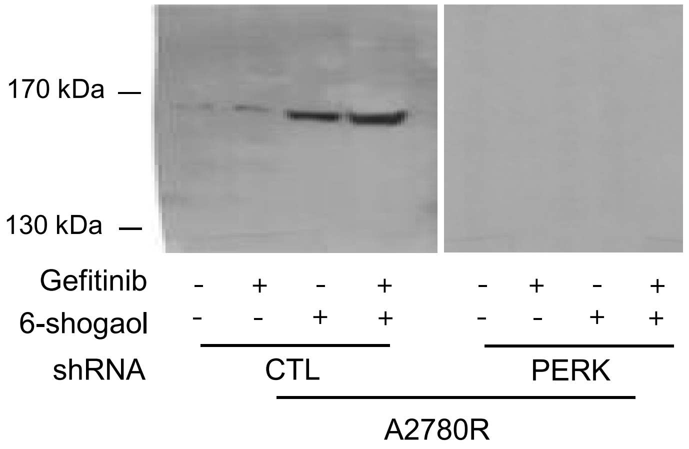

PERK (140 kDa)

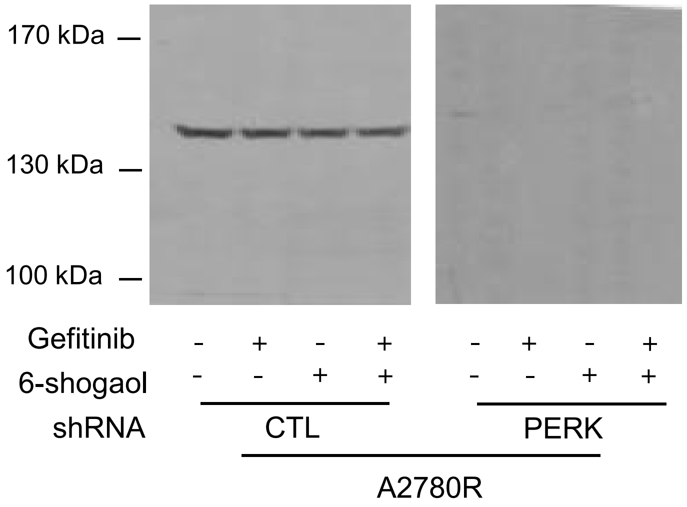

CHOP (27 kDa)

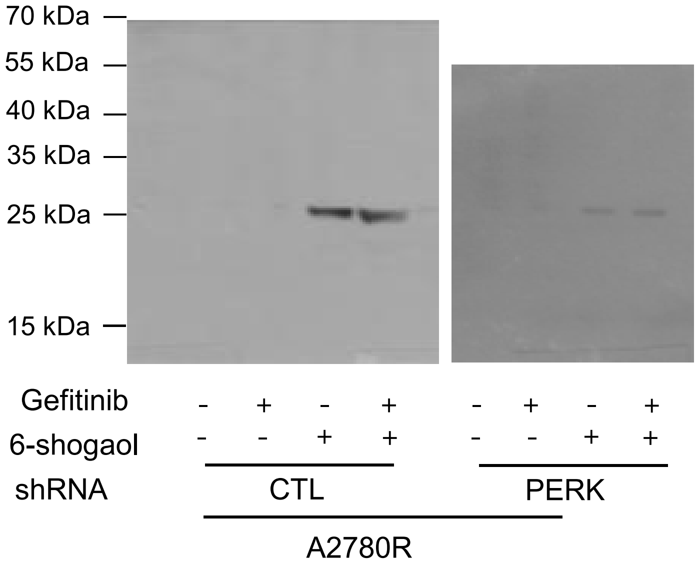

Cleaved caspase-3 (17-19 kDa)

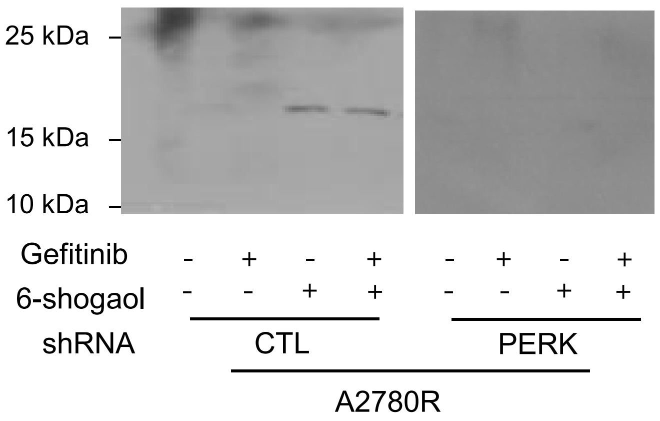

Supplement: Supplementary file 1 [file ijms-24-02639-s001.zip › ijms-2128593-supplementary.pdf]
